# Treasures from the Ocean: Deciphering the Therapeutic Utility of Marine-Derived ACE-Inhibitory Peptides in Cardiovascular Drug Discovery

**DOI:** 10.3390/ph19101533

**Published:** 2026-09-27

**Authors:** Nishshanke Arachchige Sachinee Navodya Sathsarani, Mi-Jin Yim, Habaragoda Dewage Tharushi Udayangani Wijerathne, Kalahe Hewage Iresha Nadeeka Madushani Herath, Jeong Min Lee, Kalu Kapuge Asanka Sanjeewa, Hyun-Soo Kim

**Affiliations:** 1 Department of Seafood Science and Technology, Institute of Marine Industry, Gyeongsang National University, 2-9, Tongyeonghaean-ro, Tongyeong-si 53064, Republic of Korea; sachininavodya601@gmail.com (N.A.S.N.S.); tudayangani14@gmail.com (H.D.T.U.W.); 2 National Marine Biodiversity Institute of Korea, Seocheon 33662, Republic of Korea; mjyim@mabik.re.kr (M.-J.Y.); lshjm@mabik.re.kr (J.M.L.); 3 Department of Biosystems Engineering, Faculty of Agriculture and Plantation Management, Wayamba University of Sri Lanka, Makandura 60200, Sri Lanka; madushaniherath001@gmail.com; 4 Department of Biosystems Technology, Faculty of Technology, University of Sri Jayewardenepura, Homagama 10200, Sri Lanka

**Keywords:** marine-derived peptides, ACE-inhibition, medicinal chemistry, enzyme hydrolysis, cardiovascular drug discovery, molecular docking, artificial intelligence

## Abstract

Marine-derived bioactive peptides show significant promise as candidates for cardiovascular drug discovery because of their diverse biological activities and ACE-inhibitory activity. This review outlines the current state of knowledge on marine-derived ACE-inhibitory peptides as potential therapeutic targets for cardiovascular drug discovery. This narrative review was conducted using Scopus, PubMed, and Web of Science databases with a focus on eligible studies on ACE inhibition; peptide sequence and structural characteristics; hydrolysis and purification approaches; molecular docking; pharmacokinetic strategies; drug discovery; drug-delivery approaches; and clinical studies. This review shows that ACE-inhibitory peptides are derived from marine organisms. The reviewed studies report peptide activity determined by amino acid composition, peptide length, hydrophobicity, hydrolysis method, fractionation, chromatographic purification, and characterization techniques. Computer-based molecular mechanism studies further demonstrate that marine peptides interact with ACE through hydrogen bonding, hydrophobic interactions, and interactions at the catalytic subsite surrounding the Zn ion center. Most of the peptides have demonstrated antihypertensive effects in animal models, while limited clinical evidence suggests potential for further development. This review concludes that marine-derived ACE-inhibitory peptides are promising targets for cardiovascular drug discovery. Clinical translational barriers, industrial-scale production, clinical validation, and regulatory approval are essential to address in future work. Integration of molecular docking, advanced delivery approaches, medicinal chemistry, AI-assisted peptide design, sustainable green extraction, and clinical research could expedite their translation into pharmaceutical approaches for further drug development.

## 1. Introduction

Cardiovascular diseases (CVDs) constitute a primary category of non-communicable diseases and significantly impact morbidity and mortality worldwide [1]. According to the World Health Organization (WHO), the number of people living with hypertension (systolic blood pressure ≥ 140/90 mmHg) has doubled over the past three decades, from 650 million to 1.3 billion [2]. The global prevalence of rampant hypertension remains exceptionally high at 54.6%. Around 46% of hypertensive people are totally unaware of their situation, and only 21% have their blood pressure effectively controlled with medications [3].

Beyond mortality, the socioeconomic impact is also changing. For instance, the direct and indirect economic costs of managing CVD have risen to 414.7 billion dollars annually, driven heavily by hospitalizations for myocardial infarction and heart failure [4]. As the global population ages and metabolic comorbidities, including obesity, chronic kidney disease, and type 2 diabetes, are integrated into complex clinical phenotypes such as the newly recognized cardiovascular–kidney–metabolic syndrome, the global burden of circulatory failure is projected to continue to accelerate over the coming decades [5].

In this context, various medical systems, including allopathic, Ayurvedic, Traditional Chinese, and Unani medicine, are used worldwide for the management of hypertension. In addition, both pharmacological agents are correspondingly mentioned as first-line treatments [6]. Moreover, several antihypertensive drugs, such as beta blockers, direct renin inhibitors, calcium channel blockers (CCBs), and diuretics, are used [7]. Beta blockers are considered for first-line use, but this is contentious as they are less effective than other drugs for high blood pressure. CCBs inhibit calcium influx into vascular smooth muscle, dilate arteries, enhance fibrinolysis, reduce inflammatory markers, and inhibit muscle proliferation. For instance, amlodipine reduces blood pressure while increasing nitric oxide levels [8]. ARBs reduce vascular tone by initiating endothelial nitric oxide synthase (eNOS)-derived nitric oxide [8]. Currently, the pharmaceutical industry is increasingly focusing on the marine biosphere for drug discovery because terrestrial natural product discovery is less efficient and structural duplication is high. Typically, 70% of the Earth is covered by the ocean, which contains approximately 80% of global biological diversity and acts as a dynamic chemical repository [9]. Contrary to terrestrial organisms, marine organisms synthesize unique secondary metabolites and peptide scaffolds as adaptations to extreme physical and chemical conditions. For example, piezophiles, which are found in the deep sea at pressures exceeding 1000 atm, can survive without losing the fluidity of standard biological membranes or denaturing proteins. As a result, deep-sea organisms synthesize highly compact, folded peptide structures with distinctive intramolecular disulfide networks and protein-rich domains to withstand physical compression [10].

Marine organisms can also survive in extreme thermal environments, with temperatures ranging from 2 °C to 350 °C, including Arctic environments. Organisms surviving in these zones synthesize unique peptides to maintain structural flexibility, such as thermophilic peptides that resist thermal unfolding and psychrophilic peptides that reduce the activation energy for catalytic binding. As a result, these features can be used in cardiovascular drug design to develop stable peptide therapeutics [11].

Marine organisms that live in hypersaline environments with fluctuating salt concentrations produce hydrophilic peptides rich in charged amino acids such as arginine, lysine, aspartic acid, and glutamic acid to maintain osmotic balance. These peptides form hydration shells that can bind water and tolerate hyperosmotic conditions. In cardiovascular medication, these peptides are effective for mitigating the aggregation of therapeutic peptides in human blood plasma [11].

Approximately 60% of bioactive compounds are derived from marine sources and exhibit potent antioxidant, anti-diabetic, anti-inflammatory, anticoagulant, and anticancer activities [12].

The 20 amino acids of peptides are isolated primarily by enzyme hydrolysis, microbial fermentation, and simulated gastrointestinal digestion [11]. Marine proteins offer pharmaceutical value through biologically active peptides, in addition to their nutritional value. These multifunctional peptides play crucial roles in facilitating digestion, food processing, and enzymatic hydrolysis by deleterious protein–protein interactions. Bioactive peptides commonly differ in composition and structure, including their amino acid sequences [13]. Although marine peptides can be synthesized naturally, artificial peptides have certain limitations and challenges. Limitations include the lack of scalable production, limited understanding of the peptides’ gastrointestinal stability, limited knowledge of their mechanisms of action, and inadequate clinical trials to support health claims [8].

Marine proteins have attracted interest in the pharmaceutical industry for tissue engineering, drug delivery, and microcarrier development. Fish-derived gelatin, sourced from various marine organisms, is used in the capsule industry for oral drug delivery. Marine collagen improves drug delivery, wound healing, and tissue regeneration due to its superior properties compared with mammalian collagen [11]. Unlike proteins, marine peptide-rich drugs and their derivatives are promising candidates that bridge the gap between small-molecule and large-molecule drugs. Additionally, marine-derived peptides promote high target specificity and potency through multipoint interactions with cellular receptors and enzyme active sites, resulting in nanomolar potency and reduced off-target binding. Moreover, peptides have low toxicity and accumulation because they degrade into non-toxic amino acid components [11].

Peptides can enhance angiotensin-converting enzyme (ACE) activity by altering enzyme structure and disrupting substrate recognition, suggesting a new therapeutic avenue [14]. Structural studies using nuclear magnetic resonance (NMR), X-ray crystallography, and molecular docking have revealed molecular interactions between marine peptides and ACE, including binding modes, key amino acid residues, and structural requirements for effective ACE inhibition [15]. Converting marine peptide research into clinical applications could provide safer, more effective alternatives to synthetic ACE inhibitors in CVD management. Preclinical and clinical research reveal that some marine-derived peptides can regulate blood pressure and improve CV function in animal models and humans. These findings underline the therapeutic ability of marine peptides as an alternative solution to traditional ACE inhibitors, particularly for humans experiencing serious side effects from current medications. ACE-inhibitory peptides are an interdisciplinary drug-discovery area aimed at creating safer, targeted therapies for diseases like hypertension, improving treatment and prevention. Medicinal chemistry contributes to the pharmaceutical industry by guiding the discovery and industrialization of therapeutics that drive economic growth and create job markets and technological innovations, combining nanotechnology, bioinformatics, and artificial intelligence [16].

This review highlights marine bioactive peptides and their therapeutic potential in cardiovascular drug discovery, particularly for ACE. The review examines workflows for isolating, characterizing, and identifying cardioprotective peptide sequences, then evaluates the molecular docking characteristics that govern peptide interactions with key cardiovascular targets, pharmacokinetics, gastrointestinal stability, drug-delivery considerations, and clinical status.

## 2. Method

### 2.1. Search Period and Database Selection

This study was conducted as a narrative review to synthesize studies on marine-derived ACE-inhibitory peptides and their potential contributions to cardiovascular drug discovery. The review focused on several key aspects, including marine sources of ACE-inhibitory peptides and methods for peptide isolation, purification, and identification; structure–activity relationships and molecular interactions with ACE. Moreover, the literature was critically synthesized to identify future advances that could advance the development of marine-derived ACE-inhibitory peptides as potential therapeutic targets.

In addition, this review used electronic databases, including Scopus, PubMed, and Web of Science, and drew on relevant studies published over 10 years (2016–2026) in Figure 1. The final database search was performed from December 2025 to August 2026. Key terms were used to search the articles, including: “global burden”, “marine-derived bioactive peptides”, “invertebrates”, “vertebrates”, “shellfish”, “seaweed”, ACE inhibition”, “ACE inhibitors”, “hypertension”, “cardiovascular disease”, “renin–angiotensin system”, “enzyme hydrolysis”, “purification”, “characterization”, “ultrafiltration”, “peptide structure”, “in vivo studies”, “molecular docking”, “pharmacokinetics”, “bioavailability”, “drug delivery”, “medicinal chemistry”, “structure–activity relationship”, “cyclization”, “chitosan based nanoparticles”, “oral delivery”, “quantitative structure activity relationship”, “PEGylation”, “N-terminal modification”, “Discovery Studio”, and ”in silico pipeline”.

### 2.2. Study Selection and Eligibility Criteria

Studies were considered eligible when investigated bioactive peptides and peptide-enhanced protein hydrolysates were derived from marine organisms and the study assessed their ACE-inhibitory activity regarding cardiovascular drug discovery. Review articles, systematic reviews, and meta-analyses were also examined to identify major studies and to provide a broad overview of related data on marine-derived peptides, ACE inhibition, cardiovascular mechanisms, and therapeutic enhancement.

The study was considered appropriate when it provided relevant information for the review outcomes. Therefore, studies were considered eligible when they reported at least one relevant outcome (inclusion criteria), as follows:

1. ACE-inhibitory mechanism and the percentage of marine resources;
2. IC_50_ values of ACE inhibition in the marine bioactive peptides;
3. Identification of relevant ACE-inhibitory peptide sequences;
4. Structural characteristics of active peptides;
5. Protein hydrolysis conditions, fractionation, purification, chromatographic methods;
6. Drug discovery and drug-delivery approaches;
7. Molecular mechanisms, including molecular docking findings;
8. Pharmacokinetic characteristics;
9. Antihypertensive effect in animal clinical studies and trials.

The studies were excluded (exclusion criteria) and ignored based on the following characteristics:

1. Non-peptide marine compounds without relevant data on ACE inhibition;
2. Terrestrially derived peptides with irrelevant information concerning marine-derived peptides.;
3. Insufficient information regarding the marine sources and experimental approach;
4. Irrelevant to marine organisms and marine-derived protein resources;
5. Duplicate publications and secondary documents of an already existing study without additional relatable information;
6. Publications that have unavailable relevant full texts and sufficient methodological information.

### 2.3. Screening Process and Bibliometric Analysis

The study involved two stages: first, screening titles and abstracts for relevance to marine bioactive peptides, ACE inhibition, generation, and drug discovery and second, assessing full texts of eligible studies against criteria. Full-text review articles and research articles were excluded when they did not include the requirements concerning marine sources and peptides, protein hydrolysate-derived materials, biological activities, and study-relevant methodological information required for the review. The primary reason for exclusion was noted for each study after full-text assessment.

Study selection and screening were conducted by S.N.S. independently according to the predefined exclusion and inclusion criteria. The authors resolved disagreements about study eligibility through discussion and consensus.

## 3. Physiological Mechanism of the Renin–Angiotensin–Aldosterone System (RAAS)

ACE is a zinc-dependent dipeptidyl carboxypeptidase that acts as a central regulator of systemic blood pressure through two opposing biochemical pathways: the renin–angiotensin system (RAS) and the Kallikrein–Kinin System (KKS) [16]. Moreover, ACE cleaves the C-terminal dipeptide of inactive Angiotensin I, producing Angiotensin II, a potent vasoconstrictor in the RAS. In parallel, in KKS, ACE inactivates bradykinin, a major endogenous vasodilator [17].

Physiologically and biomedically, hypertension may arise from a chronic abnormality in the brain’s RAS [17,18]. RAAS is a hormonal system that regulates blood pressure, while the baroreflex is a short-term response to sudden changes in blood pressure [19]. RAAS is responsible for long-term regulation. The RAAS pathway originates in the kidneys, where specialized juxtaglomerular cells in the walls of the afferent arterioles produce and store prorenin [19]. When blood pressure drops, prorenin is converted to renin. The body eventually releases the produced renin into the blood. Then, renin binds to angiotensinogen, a plasma protein produced in the liver, creating a decapeptide (angiotensin I), 10 amino acids long. The further conversion of Angiotensin I yields an octapeptide (angiotensin II). This conversion of Angiotensin I to Angiotensin II is a potent vasoconstrictor that increases blood pressure and stimulates aldosterone release, thereby increasing blood volume and pressure [20].

ACE is a peptidase that converts Angiotensin I into Angiotensin II [21]. ACE is normally found in the blood, the capillaries of the lungs, and the kidneys [21]. It is also known as kininase II and peptidyl-dipeptidase A. Additionally, ACE I and ACE II are the main subtypes expressed in different tissues. The balance between these two ACE types is crucial for cardiovascular and systemic stability. ACE acts as a potent vasoconstrictor with pro-inflammatory, proliferative, and pro-fibrotic effects [14]. ACE I is recognized as an exopeptidase widely expressed on the plasma membranes of vascular endothelial cells, primarily in the pulmonary circulation. This enzyme is also expressed in heart tissues, adipose tissue, the intestinal tract, the urogenital tract, and neuronal cells of the brain. It splits two amino acids from the dipeptide Angiotensin I (1–10), carboxy-terminal to the dipeptide, to form the octapeptide Angiotensin II (1–8). This ACE I mainly contributes to the classical pathway of RAS. The enzyme ACE II, first discovered in 2000, is a monocarboxypeptidase and structurally similar to ACE I, as their catalytic domains share 42% similarity [20]. ACE II has two substrates in the RAS: Angiotensin I and Angiotensin II. ACE II has 300-fold higher catalytic efficiency for Angiotensin II than for Angiotensin I. It is widely expressed in the lungs, kidneys, cardiovascular system, brain, and adipose tissue [16]. A disintegrin regulates membrane-bound ACE II levels, and Metalloprotease 17 (ADAM17) cleaves it, generating soluble ACE II. Angiotensin II, generated via activation of the Angiotensin I receptor, up-regulates ADAM17 activity. It induces membrane-bound ACE II and theoretically increases disease processes in the RAS. ACE II is considered the functional receptor for both severe acute respiratory syndrome coronavirus 1 (SARS-CoV-1) and severe acute respiratory syndrome coronavirus 2 (SARS-CoV-2), which caused the SARS epidemic in 2002–2004. ACE II is expressed on small intestine enterocytes and lung alveolar epithelial cells. Furthermore, ACE II contributes to the counter-regulatory (protective) pathway. Aminopeptidase A (APA) degrades Angiotensin I and Angiotensin II to a single aspartate residue from the N-terminus [22]. Moreover, aminopeptidase N converts Angiotensin III to Angiotensin IV. Angiotensin IV acts via its receptor (AT4R) and insulin-regulated membrane aminopeptidase (IRAP) [23].

Angiotensin II has a short lifetime of one to two minutes. It is degraded into angiotensin III and IV, which have weaker effects. Normally, overactive RAAS is a cause of hypertension. RAAS is a frequent target of antihypertensive drugs. As previously mentioned, ACE inhibitors and angiotensin receptor blockers are common treatments for hypertension [24]. Hypertension mainly promotes the formation of Angiotensin II, a potent vasoconstrictor (Figure 2).

### Role of the ACE-I and ACE-II Inhibition

Lack of ACE results in a significant reduction in high blood pressure, with systolic blood pressure around 73 mm Hg compared with 110 mm Hg in wild-type mice. Therefore, ACE inhibition is a recent advancement in hypertension management [25]. RAS inhibitors attenuate the neurogenic actions of Angiotensin II on peptide expression in the brain [24]. Mouse models have widely demonstrated the effectiveness of ACE inhibitors in lowering blood pressure and treating cardiovascular diseases [25].

ACE inhibitors are first-line agents for blood pressure control because they improve cardiovascular and renal outcomes by reducing hospitalizations and microalbuminuria [24]. ACE inhibitors, typically abbreviated as “pril” (for instance, captopril and lisinopril), are synthetic drugs that target ACE. Moreover, these inhibitors are categorized into three groups according to their chemical composition: sulfhydryl-containing ACE inhibitors (captopril), phosphorus-containing ACE inhibitors (fosinopril), and dicarboxylic-containing ACE inhibitors (enalapril, benazepril, lisinopril, moexipril, perindopril, ramipril, quinapril, and trandolapril) [16]. Another way of categorizing the ACE-inhibitory peptides is into true inhibitors (peptides are unable to change activity by pre-incubation), substrates for ACE (peptides can be converted to inactive peptides), and pro-drug peptides (peptides can be converted to true inhibitors by ACE/gastrointestinal proteases). Side effects of ACE inhibitors include dry cough, hypotension, dizziness, hyperkalemia, and angioedema [23]. ACE inhibitors block the body’s normal mechanism for maintaining blood pressure. ACE converts Angiotensin I into Angiotensin II. Angiotensin II can then narrow blood vessels and stimulate the release of hormones that raise blood pressure [20]. Therefore, ACE inhibitors inhibit ACE to lower rising blood pressure by reducing Angiotensin II levels. By lowering Angiotensin II, the blood vessels relax, reducing blood pressure to a normal level. This also helps the kidneys pass more salt and water into the urine. In addition, reducing body fluid volume results in a smaller blood volume for the heart to pump [20]. By relaxing blood vessels and reducing fluid levels in the blood vessels, ACE inhibitors help maintain blood pressure. This reduces the strain on the heart and arteries. This improves the heart muscle’s pumping action, thereby restoring normal blood pressure [23].

Synthetic ACE inhibitors have various side effects. Therefore, demand for natural, safe ACE inhibitors has increased. Many natural ACE inhibitors isolated from functional foods and bioresources have been identified, with improved safety and efficacy for treating hypertension [26]. Peptides derived from natural products inhibit ACE by blocking the transformation of Angiotensin I to Angiotensin II. Synthetic ACE inhibitors are more effective, despite the side effects. The side effects include dry cough, taste disturbance, kidney disease, and angioedema. Coughing occurs in 3.9% to 35% of patients across several ACE inhibitors, including perindopril. For that reason, iron supplements have been found to reduce cough scores in patients [25]. Rather than synthetic ACE inhibitors, ACE-inhibitory peptides derived from various food sources are emerging as alternatives because of their bioavailability and lack of side effects [27].

## 4. Bioprospecting and Drug Discovery Architecture from Marine Peptides

### 4.1. Marine Sources of ACE-Inhibitory Peptides

#### 4.1.1. Invertebrates

Marine invertebrates, including sponges, mollusks, gastropods, tunicates, and echinoderms, are a diverse biological resource for bioprospecting. These organisms have evolved complex chemical defense mechanisms over millions of years to survive in competitive marine environments. Their survival depends on producing unique secondary metabolites and innate multi-component venoms [25,27,28].

Sponges contain high levels of structurally modified peptides, including cyclic peptides and depsipeptides (peptides with one or more ester bonds in place of amino acids) that often feature unique structural elements such as nonproteinogenic and D-amino acids. These structural features are crucial for drug discovery because steric hindrance protects molecules against enzymatic degradation by exogenous proteases in human plasma. This defense results in sponge-derived peptides having extended serum half-lives and improved metabolic stability compared to peptides from terrestrial sources [29]. In the context of vascular health, the molecules inhibit endothelial cell adhesion and block vascular smooth muscle cell proliferation, thus counteracting atherosclerotic plaque progression. The peptide KENPVLSLVNGMF derived from marine sponges exhibited cytotoxicity against the human cancer cell line (HeLa) in a dose-dependent manner, and its cytotoxicity (EC_50_ of 0.67 mM) was 3.8-fold higher than that of 5-FU (EC_50_ of 2.56 mM) [29].

Marine gastropods, including cone snails (*Conus magu*), deliver a potent venom containing conotoxins (ω-Conotoxin MVIIA), and these are disulfide-rich oligopeptides with rigid, stable 3D structures. Although known for neuropharmacological effects, these conotoxins also have significant cardiovascular applications [30]. Marine bivalves and tunicates consist of low-molecular-weight oligopeptides, typically with 2 to 9 amino acids. These low-molecular-weight peptides can bypass the spatial constraints that the active sites of larger proteins face when binding to target enzymes. Peptides from these organisms show highly competitive binding affinities for ACE. Hydrophobic C-terminal residues were introduced into the ACE active site, aligning with and inactivating the central catalytic zinc ion. Hence, this mechanism mimics the therapeutic effects of synthetic ACE inhibitors while reducing side effects [31].

Shellfish, which are mollusks, and invertebrates such as snails, razor clams, oysters, squid, mussels, and octopus, are also rich in ACE-inhibitory peptides. The antibacterial peptide (VTVP) and the IC_50_ value of 125.58 µM for the ACE-inhibitory peptide (KSHPH) were isolated from Kuruma shrimp (*Marsupenaeus japonicus*) [32]. The major ACE-inhibitory peptide was produced with the highest ACE-inhibitory activity (IC_50_ = 76.8 ± 15.2 µg/mL) via alcalase hydrolysis of a cuttlefish industrial manufacturing effluent. However, the study did not identify a specific peptide with antihypertensive activity, and the IC_50_ values of the peptide fractions ranged from 1.92 to 8.83 µg/mL [32].

Sea cucumbers are potential aquaculture products, particularly in Asian regions, for medicinal and culinary applications. Usually, ACE-inhibitory peptides have been found in hydrolysates of sea cucumber gonads after large-scale purification of active peptides [33]. Three noncompetitive inhibitory peptides, HDWWKER, DDQIHIF, and THDWWKER, with IC_50_ values of 333.5 µmol/L, 5863.6 µmol/L, and 1291.8 µmol/L, respectively, were isolated from the gonads of the sea cucumber for further study due to their favorable effects. HDWWKER could significantly decrease the systolic blood pressure of spontaneously hypertensive rats (SHRs) [34,35].

#### 4.1.2. Vertebrates

Beyond the nutritional value, proteins mediate a diverse array of physiological processes, fulfilling catalytic, structural, and locomotor functions in the body [36]. Furthermore, the body can quickly digest and absorb fish proteins, yielding hydrolysates with functional bioactivity. Fish proteins offer novel avenues for developing biotechnological products with antimicrobial, antioxidant, and antihypertensive effects [37]. Marine-derived fish protein enzyme hydrolysates (FPHs) are obtained from several edible fish species, including mackerel, which exhibits antioxidant activity. Lysine, methionine, and ACE-inhibitory peptides have been found in sardine, shellfish, and salmon [35]. Fish muscle normally contains high levels of amino acids such as glutamic acid, taurine, proline, glycine, alanine, and arginine. Fish are rich in taurine, which is involved in mammalian development and has been shown to reduce blood pressure, enhance CV performance, and lower blood cholesterol levels. White fish typically have more taurine than oily fish, as confirmed by European specialists [38]. Moreover, the bioactivity of fish hydrolysates was contributed by methionine, tryptophan, tyrosine, cysteine, and phenylalanine. Taurine enhances central nervous system protection by reducing endoplasmic reticulum stress and acting as an antagonist at neurotransmitter receptors [38].

Waste salmon bones from the fishing and fish-processing industries can yield various bioactive peptides, including those derived from collagen, gelatin, and amino acids. The FCLYELAR peptide was identified as an uncompetitive ACE inhibitor, attributed to hydrogen-bond interactions with ACE [36].

#### 4.1.3. Marine Macroalgae

Marine algae-derived peptides are widely studied for their potential as natural ACE inhibitors. Rather than fish-derived peptides, marine algae-derived ACE-inhibitory peptides have high IC_50_ values. For instance, trypsin hydrolysis of proteins from sea lettuce, *Ulva intestinalis* and *Gracilariopsis lemaneiformis* has been found to yield high-ACE-inhibitory peptides with IC_50_ values of 219.35 µmol/L and 474.36 µmol/L, respectively [37]. Table 1 presents data on marine organisms that produce a variety of bioactive proteins and peptides.

## 5. Modern Isolation and Identification Strategies

The discovery of the marine-derived ACE-inhibitory peptides generally involves peptide generation, fractionation, purification, and structural identification and characterization. The preparation of ACE-inhibitory peptides, including extraction and purification from marine organisms, is a complex process that improves peptide yield and bioactivity while preserving their structural integrity [56]. Figure 3 illustrates the sequential workflow involved in the production and characterization of ACE-inhibitory peptides.

This multi-step process begins with preparing the raw marine sample material. First, the obtained sample undergoes cleaning, defatting, and the removal of unnecessary components. Then, the proteins are primarily extracted with various solvents, such as acetic acid, to ensure complete dissolution while preserving their integrity. Filtration is used to separate soluble from insoluble proteins [59]. The soluble proteins are then precipitated by salt (e.g., NaCl) through centrifugation to increase protein concentration. The precipitated proteins are hydrolyzed, breaking down into small peptide fragments [60].

Various methods, including enzymatic, thermal, and bacterial fermentation, can be used to hydrolyze proteins. This alters the physicochemical and techno-functional properties of the protein. Digestive proteases (trypsin, pepsin, and chymotrypsin) and commercially available enzymes (papain, bromelain, and alcalase) are used for enzyme hydrolysis [61]. Enzyme hydrolysis is the predominant method for obtaining antihypertensive peptides from natural resources because of the enzyme’s specificity and effectiveness in targeting bioactive peptides [62]. For the production of ACE-inhibitory peptides, consider only high digestibility, not other factors, for commercialization. Commercially available food-grade proteases, including alcalase, protamex, and flavourzyme (from microorganisms); papain and bromelain (from plants); and trypsin, pepsin, and chymotrypsin (from animals), are commonly used for enzyme hydrolysis. The crude proteases isolated from the visceral mass are beneficial for protecting the environment from pollution and for generating economic profit. Depending on the specific enzyme–substrate combination, the functional end products are determined. The substrate specificity of proteases enhances the peptide profile and biological functionality of the protein hydrolysates. The amino acid composition of precursor proteins and the structural characteristics of peptide bonds also guide the selection of a suitable enzyme for preparing a bioactive peptide with a specific function [62,63]. Table 2 summarizes several marine peptides recognized as exhibiting ACE-inhibitory properties.

The prepared sample is purified using several techniques, including high-performance liquid chromatography (HPLC), membrane separation, ion-exchange chromatography, gel permeation chromatography (GPC), and ultrafiltration. However, the separation and purification of ACE-inhibitory peptides from the hydrolysis products is a bottleneck due to the complexity and high cost involved. To address these issues, an immobilized metal affinity medium (IMAM) is a useful strategy for rapidly isolating ACE-inhibitory peptides from protein hydrolysates. The purified peptides are subsequently characterized by mass spectrometry, enabling structural elucidation of peptides exhibiting potency comparable to that of naturally occurring ACE-inhibitory peptides [57,64].

Furthermore, the purification enables correct attribution and characterization of the peptide. Therefore, the identification of a specific peptide represents a crucial step from a bioactive marine peptide to a potential therapeutic target. After the peptide is obtained, its activity can be further investigated through structure–activity relationship studies, molecular docking, peptide synthesis, gastrointestinal stability assays, clinical trials, and pharmacokinetic evaluation [32]. From this perspective, the generation and identification of the peptide are not merely analytical steps but constitute an essential part of the marine peptide drug discovery pipeline. It also assesses the recovery and characterization of individual peptides, including outlined structures and biological activities, for subsequent pharmaceutical evaluation [77].

### Biotechnological Manufacturing Approaches

Beyond direct extraction from marine organisms, biotechnological manufacturing can produce these peptides by identifying specific ACE-inhibitory peptide sequences. Moreover, recombinant production is a potential strategy for producing ACE-inhibitory peptides, as supported by similar experimental evidence [78]. In this strategy, suitable microbial cells such as *Escherichia coli* could express the corresponding peptide-linked sequences as a production host rather than the original marine organism [79]. In this context, dependence on continuous extraction of marine biological resources may be reduced, and production and quality control can be made more uniform. The low molecular weight peptides can be difficult for microorganisms to produce due to rapid degradation by enzymes that are produced, and the amount produced may be very low. Hence, researchers often use DNA to produce multiple copies of the same peptide sequence, rather than producing a single copy. Recombinant DNA technology could also be used to produce modified versions of the marine peptides [80]. For instance, the marine-derived ACE-inhibitory peptide LYPVK was produced in *E. coli* BL21 (DE3) using a synthetic DNA construct encoding tandem repeats, a DNA sequence designed to produce multiple copies of the same peptide. This peptide was expressed as a larger recombinant protein with a His-tag, purified, and then released using trypsin [81]. Comparably, another ACE-inhibitory peptide was produced in *E. coli* using a DNA construct containing six tandem repeats of the peptide sequence. These six copies helped to protect the peptide from degradation inside the host cells [82].

## 6. Drug Discovery: Marine ACE-Inhibitory Peptides

### 6.1. Structural Insights and Structure–Activity Relationship (SAR) of ACE-Inhibitory Peptides

Bioactive peptides with ACE-inhibitory capabilities exhibit an exceptionally broad and diverse structural design, differing significantly by raw material source and processing method. Peptides from terrestrial animal sources are often derived from proteins encoded in milk, such as lactotripeptides from caseins, hemoglobin from bovine blood fractions, royal jelly, and gelatin. Peptides from terrestrial plant sources are abundant in the seed storage proteins of soy, wheat, chickpeas, and numerous agricultural by-products [31]. Likewise, marine organisms are broadly sourced from fish collagen, mollusks, sponges, and marine algae.

These marine peptides mostly possess unique structural configurations that confer high affinity and very low IC_50_ values [63,83]. The amino acid composition, sequence configuration, and absolute chain length dictate the effectiveness of an ACE-inhibitory peptide. Advanced in silico profiling and quantitative structure–activity relationship models have defined several core structural rules. Additionally, chains of 2 to 20 amino acids show high ACE-inhibitory activity [30]. The short fragments, including dipeptides and tripeptides, bind cleanly within the enzyme’s narrow active-site clefts [26,31].

#### 6.1.1. Complete Chemical Structures and Stereochemical Features

Marine-derived ACE-inhibitory peptides commonly have 2–12 amino acids. This allows them to interact effectively with the ACE catalytic channel. Aromatic amino acids, near the C-terminus often enhance activity, as do branched-chain hydrophobic amino acids. However, those are general trends, because ACE inhibition depends on a combination of residue identity, position, conformational flexibility, and overall physicochemical properties [84].

To provide more comprehensive detail on the structural features of ACE inhibition, the complete chemical structures of representative marine-derived ACE-inhibitory peptides should be known rather than described individually by their amino acid sequences. Thus, peptides with similar amino acid compositions may show different inhibitory activities because of changes in residue sequence, stereochemistry, and 3D conformation [18,85]. For representative potent peptides, the relationship between their complete chemical structures and ACE-inhibitory activity can therefore provide vital insights into the structural determinants of inhibition. In particular, the position of hydrophobic, aromatic, and conformationally restricted residues, together with the peptide’s overall 3D shape, may influence hydrogen bonding, hydrophobic interactions, electrostatic interactions, and other non-covalent interactions within the active site [17].

#### 6.1.2. Key Amino Acid Determinants Associated with ACE Inhibition

Specific amino acids play an important role in determining the ACE-inhibitory activity of peptides. Aromatic amino acids, including phenylalanine (Phe), tyrosine (Tyr), and tryptophan (Trp), contribute through hydrophobic and aromatic interactions with residues lining the ACE catalytic channel. Leucine (Leu), isoleucine (Ile), and valine (Val) are hydrophobic branched-chain amino acids and enhance peptide–ACE interactions by increasing hydrophobic contacts within the binding pockets. Among these amino acids, proline (Pro) is the main amino acid associated with ACE inhibition because of its conformational rigidity. It can also influence peptide conformation and allow favorable interactions within the ACE active site. Moreover, positively charged amino acids such as arginine (Arg) and Lysine (Lys) may contribute to electrostatic interactions with negatively charged amino acids in the active site. Therefore, ACE-inhibitory potency is determined by the combined effects of amino acid identity and sequence position [86].

The position of an amino acid is also a primary factor, as is its identity. Molecular interaction studies suggest that ACE-inhibitory peptides frequently contain hydrophobic, positively charged, and aromatic amino acids at specific positions with respect to the C-terminus. In particular, the C-terminal region strongly influences ACE recognition, as its terminal residues interact with ACE binding pockets; potent inhibitors often contain Pro, Phe, Tyr, and other amino acids. Proline is considered a particularly important C-terminal amino acid in ACE-inhibitory peptides, although its contribution depends on the complete peptide around the determinants. Lysine and arginine may also contribute to ACE recognition through electrostatic interactions, while their position within the sequence can influence the peptide’s orientation and binding mode [87].

Specifically, ACE-inhibitory activity should be interpreted based on the collective structural characteristics of the entire peptide rather than an amino acid individually. In addition, the amino acid composition, sequence, terminal residues, hydrophobicity, and charge distribution are key determinants of the structure–activity relationship of ACE-inhibitory peptides [47]. Typically, ACE inhibition for all peptides could not be predicted by the universal sequence motif. Several structural characteristics are repeatedly associated with ACE-inhibitory activity [88].

#### 6.1.3. Peptide Conformation and ACE Binding Interactions

Beyond the two-dimensional (2D) chemical structure, the 3D shape of a peptide is of vital significance to biological activity. Peptides are conformationally flexible molecules that can adopt different spatial arrangements depending on their amino acid sequence, stereochemistry, intramolecular interactions, and surrounding nature. The resulting conformation defines the spatial distribution of hydrophobic, aromatic, charged, and hydrogen-bonding regions and therefore improves their ability to interact with the ACE-catalytic channel [89]. Recent studies on marine-derived ACE-inhibitory peptides have emphasized that their unique 3D features contribute to ACE inhibition and that structure-based approaches can improve molecular perception of peptide–ACE interactions [63,86].

Peptide conformation may range from relatively planar arrangements to more folded structures. Additionally, planar geometry should not be considered inherently more favorable than a folded conformation. Rather, biological activity depends on whether the adopted conformation positions the relevant functional groups in an orientation that corresponds to the ACE binding site. A folded peptide may convey vital residues into close spatial proximity and create a promising interaction surface. In addition, an extended peptide may expose aromatic amino acids that can interact with specific regions of the enzyme [86]. Thus, two peptides with similar amino acid compositions may display substantially different ACE-inhibitory activities due to changes in their 3D conformations. Because of this, proline is particularly relevant because its aromatic structure restricts backbone flexibility [90]. Correspondingly, the positions of hydrophobic and charged amino acids within a peptide can affect intermolecular interactions and the preferred peptide conformation. Ultimately, the structural features can determine whether critical amino acids are appropriately oriented for integration with the ACE catalytic pocket [91]. When considering stereochemistry and conformation, the configuration of chiral amino acids governs the spatial orientation of their side chains and influences the available conformational space of the peptide. Therefore, stereochemical changes determine the peptide’s 3D shape and its ability to fit into the ACE active site. The changes may affect interactions between the peptide and ACE. [92]

### 6.2. Interaction of Isolation and Computational and Molecular Mechanisms of ACE Inhibition

Isolation of marine peptides is vital for identifying specific ACE-inhibitory peptides from marine protein hydrolysates. Although crude hydrolysates contain multiple active peptides, successive fractionation and purification techniques are used to characterize individual ACE-inhibitory sequences and hydrophobic and aromatic amino acids, in specific positions and combinations within peptide sequences. For instance, four ACE-inhibitory peptides identified from the same sea cucumber (*Acaudina molpadioidea*) hydrolysate demonstrated different inhibitory activities rather than having similar IC_50_ values. PNLG and PNVA are both tetrapeptides with similar molecular weights (399.45 Da) but different IC_50_ values. PNLG and PNVA have IC_50_ values of 13.16 and 8.18 µM, respectively. Those different IC_50_ values indicate that amino acid identity and sequence arrangement can improve ACE-inhibitory activity beyond molecular weight alone [93]. VYAP, VIIF, MAW, GIHETTY, EKSYELP, and VELYP peptides observed from cuttlefish (*Sepia officinalis*) have different IC_50_ values of 6.1, 8.7, 16.32, 25.66, 14.41 and 5.22 µM, respectively. VELYP pentapeptide has a lower IC_50_ than the VYAP tetrapeptide, demonstrating that peptide length and structural characteristics can strongly influence the activity [84]. Alaska pollock (*Theragra chalcogramma*) fish peptides GPL and GPM have IC_50_ values of 2.6 and 17.3 µM, respectively. These fish-derived tripeptides, which replace only the C-terminal Leu along with Met, significantly and strongly change the reported activity [84]. Furthermore, direct comparison of IC_50_ values requires caution because bioassay conditions can influence the measured inhibitory potency. The source concentration, ACE preparation, substrate used for ACE activity measurement, assay method, and incubation conditions may affect the IC_50_ results. Integrating peptide purification with computational analysis, specifically molecular docking, allows assessment of active-site interactions and helps identify residues potentially responsible for binding. Thus, modern isolation strategies for marine peptides are the initial step in the drug discovery process. A purified peptide provides the basis for further molecular docking, structure–activity studies, stability evaluation, and in vivo assessment of hypertension [94]. Following peptide isolation and purification, molecular docking is a powerful method for understanding the interactions between ACE-inhibitory peptides and the ACE enzyme. Common tools, including AutoDock, MOE, Glide, and Discovery Studio, can predict peptide binding poses and assess interactions, with Discovery Studio, in particular, being used for visualizing and characterizing peptide–ACE interactions [95]. Docking predicts hydrogen bonding, hydrophobic, electrostatic, and van der Waals interactions, in addition to interactions between binding subsites including S1, S2, and S1′ and the catalytic Zn^2+^ center [96].

For instance, researchers used molecular docking to study the WAR peptide, derived from monkfish, and found that it forms multiple hydrogen bonds with ACE active site amino acids (Ala354, Glu384, His353, and Lys511). This suggests a stable binding conformation and potent ACE-inhibitory activity. WAR also engaged ACE residues through hydrogen bonds and hydrophobic interactions, particularly with Val518 and Thr701. The DS 2017 R2 Client predicted the binding sites for ACE-WAR and ACE-WQR. Bonding energies stabilized the ACE-peptide. The docking of stabilized ACE-WAR and ACE-WQR resulted in CDOCKER ENERGY values of −93.322 and −98.5874 kcal/mol, respectively, indicating a good binding affinity between both peptides and ACE. This indicates strong binding affinity and a non-competitive inhibitory mechanism [97].

An in silico pipeline is a computational simulation and predictive model for rapidly identifying and filtering genetic variance in a virtual environment [98]. Rather than relying on traditional methods, recent studies prioritize the high-value exploitation of seafood processing by-products, coupled with novel, progressive in silico pipelines. In vitro screening has steadily identified the precise chemical and structural determinants that affect optimal ACE binding [99]. Amino acid positioning is a key determinant: targeted binding capacity increases when hydrophobic amino acids are distributed at the N- and C-terminal positions [100]. The other determinant is molecular weight: the fraction has a molecular weight of less than 10 kDa. It consists of short-chain oligopeptides with molecular weights under 1 kDa and fewer than 20 amino acids, which indicate high in vitro ACE-inhibitory activity [100].

Modern analytical integration facilitates the standard timeline for isolating seafood peptides. Recent studies rely on an integrated pipeline that describes the modern computer-to-bench workflow (Figure 4) [101]. The advanced liquid chromatography–high-resolution mass spectrometry (LC-HRMS) linked to databases, including BIOPEP, facilitated the rapid determination of cleavage sites and bioactivities. Moreover, molecular docking and molecular dynamics simulations were performed to determine the peptide’s physical stability, hydrogen-bonding networks, and binding free energy within the human ACE active site [100].

For instance, LRDGEIILR (LR-9) from *C. formosanum* has an IC_50_ of 94.28 ± 1.84 µM; inhibition kinetics revealed it to be a competitive inhibitor. Molecular docking predictions showed that LR-9 interacts preferentially with critical residues in the ACE active site. LR-9 does not directly interact with Zn^2+^ at the active site but instead binds to the S1 active-site pocket, suggesting it is a potential competitive inhibitor. The binding energy of LR-9 was −160.281 kJ/mol, indicating a strong binding affinity to ACE. This strong affinity suggests LR-9 can effectively compete with natural substrates or inhibitors for binding to ACE. The CDOCK interaction energy of LR-9 was 130.256 kJ/mol, further indicating a strong and favorable interaction with ACE. Moreover, toxicity prediction indicated that LR-9 is non-toxic. As a result, the ACE I peptides of *C. formosanum* can be used in functional food and drug development [102].

Molecular docking showed that four peptides from deep-sea mollusks interact differently with the active pocket, with LAAHFAR forming the most hydrogen bonds with key residues like Glu162 and Asp377. This dense interaction network stabilizes LAAHFAR, improving its capacity to interfere with substrate access to the Zn^2+^-containing catalytic motif. FTTFGK, NGAGPYGRP, and YAAPYR formed fewer hydrogen bonds and fewer interactions with catalytically essential acidic residues than LAAHFAR. LAAHFAR forms the most hydrogen bonds, including key interactions with Glu162 and Asp377. It is essential for ACE catalysis, and biochemical assays confirm it has the strongest ACE-inhibitory activity due to its superior engagement with key residues [103].

In particular, computational bioassay analysis of marine-derived ACE-inhibitory peptides has recognized hydrogen bonds and hydrophobic interactions with amino acids located around the ACE active site [63]. In some cases, interactions around the catalytic center contribute to ACE inhibition. Conversely, docking scores explain mechanisms but should be evaluated with experimental ACE-inhibitory activity, not taken as direct potency measurements [98]. The relationship between predicted binding and experimental IC_50_ values is crucial for structure–activity relationships. Peptides with lower values form more interactions, show stronger hydrophobic complementarity, and adopt a more suitable conformation within the ACE catalytic channel, while higher values indicate weaker binding. However, docking scores may not ensure low experimental values because of conformational flexibility, peptide stability, and physicochemical conditions [63,104].

A more promising docking score generally indicates a more likely predicted interaction under a given scoring function. However, docking scores generally should not be interpreted as direct experimental determinants of ACE-inhibitory potency. In this context, computational predictions are not ideal for experimental data, including IC_50_ values. Validation can also include redocking experimentally predicted ligands and comparing determined and experimental poses using RMSD. For promising peptides, binding free-energy approaches such as GBSA may offer additional insight into the energetic influences on peptide and ACE binding [105]. Furthermore, ACE inhibitors block ACE by binding to its catalytic region, preventing the conversion of Angiotensin I to Angiotensin II. Inhibitory peptides can form multiple non-covalent bonds with amino acids around the active site as they enter the ACE catalytic channel. These interactions can stabilize the peptide within the catalytic channel and interfere with the proper positioning of Angiotensin I at the ACE catalytic site. Some peptides may interact with the catalytic Zn^2+^ region, potentially inhibiting enzyme catalysis. Thus, ACE inhibition typically depends on the peptide’s overall binding mode and structural complementarity rather than a single amino acid [63,71,106]. Based on this mode of action, ACE-inhibitory peptides should be interpreted by integrating experimental ACE-inhibitory activity with structural and computational evidence. Peptides that exhibit low IC_50_ values have promising occupation of the ACE catalytic channel and interactions. This integrated approach provides a strong basis for understanding how stereochemistry, 3D conformation, and peptide sequence influence ACE inhibition [106].

### 6.3. Peptide Optimization

Optimizing peptide structure through modifications like cyclization, PEGylation, N-terminal modification, and C-terminal amidation is crucial in drug discovery. Furthermore, these changes improve the bioavailability, stability, potency, and resistance to enzymatic degradation of marine-derived ACE-inhibitory peptides [107]. The structural modifications are as follows:

- Conversion of linear peptides into cyclic polypeptides, which contributes to increasing conformational stability and preventing enzymatic degradation [108].
- Binding of polyethylene glycol (PEG) can influence peptide stability and circulation time while reducing rapid renal clearance [109].
- Modifying the N-terminus can also improve resistance to enzymatic degradation and alter peptide stability and biological activity [110].
- Conversion of the C-terminal carboxyl group to an amide region contributes to facilitating enzymatic stability and, depending on the sequence, improves ACE-binding affinity [111].
- Sequence-based peptide engineering, such as incorporating non-natural residues, enhances potency, stability, selectivity, and pharmacokinetic properties [112].
- Quantitative structure–activity relationship (QSAR) approaches can link peptide structural features to ACE-inhibitory activity, thereby supporting the identification and optimization of promising peptides. They help determine the structural characteristics of potent peptides before experimental testing. Ultimately, it connects experimental peptide modifications with computational drug design [86].

## 7. Pharmacokinetics

Marine-derived ACE-inhibitory peptides exhibit a wide range of structural characteristics and advantages, yet are largely constrained by oral delivery barriers [113].

Enzymatic antagonism involves a ligand reducing an enzyme’s ability to interact with its substrate, thus blocking its catalytic action. Researchers typically use QSAR modeling and analyze the Michaelis–Menten equation (Lineweaver–Burk plot), which offers K_m_ and V_max_ values to indicate the inhibitory potential of peptides [113].

The absolute top speed at which the enzyme can work is when the enzyme completely breaks down the substrate, which means the maximum velocity (V_max_). The Michaelis constant (K_m_) is the number of substrates that are needed to obtain the enzyme running at half of its top speed [114].

Competitive, non-competitive, and mixed-type inhibition modes are distinct mechanisms that reduce the initial velocity of ACE. Competitive inhibition blocks the active site while increasing K_m,_ with V_max_ unchanged. The peptide also shares structural similarity with Angiotensin I and directly binds to the active-site pockets, blocking substrate entry [83]. Noncompetitive inhibition typically reduces V_max_, while the apparent K_m_ remains stable. The peptide then binds to a surface region away from the active core. It forms a dead-end complex that prevents product formation in the presence of a substrate [115]. Uncompetitive inhibition decreases both V_max_ and K_m_. The peptide binds only to the pre-existing enzyme-substrate complex, physically locking it and blocking catalytic turnover. Mixed-type inhibition modifies both V_max_ and K_m_. Because the peptide interacts with both the free enzyme and the ES complex, it typically induces structural distortion through the binding cleft [116].

Optimizing marine-derived ACE-inhibitory peptides for therapeutic use requires addressing pharmacokinetic limitations. Absorption, distribution, metabolism, and excretion (ADME) are primarily affected by molecular size, charge, peptide sequence, and hydrophobicity. Gastrointestinal proteolysis and plasma peptidases can degrade absorbed peptides, affecting their bioavailability and clearance. Small peptides can cross intestinal epithelial cells through peptide transporters, depending on their sequence. To develop marine-derived ACE-inhibitory peptides as therapeutics, optimizing their stability, exposure, and resistance to degradation is crucial [117].

Marine peptides face significant physiological challenges to clinical applications. Furthermore, the primary challenge for oral delivery of ACE-inhibitory peptides is the harsh proteolytic environment of the gastrointestinal tract. The gastric and intestinal phases are the two phases during which ingested peptides undergo enzymatic attack [108].

Digestion has two stages: the gastric phase involves acidic denaturation by pepsin, and the intestinal phase uses pancreatic proteases to break down peptides, including marine peptides, into amino acids for absorption [63].

In this context, marine-derived ACE-inhibitory peptides may have limited therapeutic application because of rapid enzymatic degradation and low oral bioavailability. To overcome these limitations, novel drug-delivery strategies are crucial to protect peptides and improve ADME [118]. Researchers have used polymeric nanoparticles, such as chitosan-based nanoparticles, to deliver ACE-inhibitory peptides. Another drug-delivery approach is liposomes, which include a lipid bilayer embedded with fat-soluble molecules that can facilitate the transport of different molecules while modifying the surface to increase stability [119]. Hydrogels can also serve as drug-delivery systems because they are 3D polymeric networks that facilitate controlled peptide release. Furthermore, pH-responsive hydrogels can degrade when environmental acidity changes. This also helps protect peptides in the stomach and promotes their release in the intestine [120].

## 8. Clinical Status

The most documented bioactive property is its antihypertensive effect via ACE inhibition. Animal models and evaluations of ACE-inhibitory peptides derived from marine organisms have been extended to in vivo studies. ACE-inhibitory peptides obtained from enzyme hydrolyses of food proteins exhibit in vitro and in vivo activity in spontaneously hypertensive rats (SHRs). However, only a small number have been tested in humans [74]. The SHR model is most commonly used in mice because of its genetic predisposition to high blood pressure, which is similar to that in humans. The Dahl salt-sensitive (DSS) rats and SHRs are established models for studying high blood pressure due to the roles of diet and genetic factors [121]. The collagenase-treated hydrolysate from 60 min of hydrolysis and the original RSGH were analyzed for ACE-inhibitory activity using in vitro gastrointestinal proteases to predict in vivo antihypertensive effects [122]. Omapatrilat is a potent constrained peptidomimetic vaso-peptidase inhibitor that inhibits ACE by antagonizing the blood pressure response to Angiotensin I in rats [74].

The QAGLSPVR and LEPVR peptides significantly reduce systolic blood pressure at a dose of 2000 mg/kg, demonstrating their potential as ACE-inhibitory peptides in rat models [123]. The antihypertensive effect of PPLLFAAL was assessed by measuring blood pressure in SHRs with tail systolic blood pressure (SBP) >180 mmHg. According to in vivo results, the molecular weight of VFSGWAA exhibited the highest ACE-inhibitory activity [70]. As shown in Table 3, most seafood-derived peptides were evaluated using spontaneously hypertensive rats (SHRs) as the animal model. Researchers also used human subjects in clinical studies to determine ACE-inhibitory activity.

However, using marine-derived ACE-inhibitory peptides in clinical use requires methodical evaluation and comprehensive characterization. Ultimately, regulatory requirements vary by the specific application, with different pathways suitable for pharmaceutical, nutraceutical, and functional food products. Therefore, effective clinical translation requires optimizing peptides, delivery technologies, preclinical validation, and regulatory approval [131].

## 9. Challenges and Clinical Obstacles in the Development of Marine-Derived ACE Inhibitors

### 9.1. Bioavailability and Gastrointestinal Stability

Translating marine-derived ACE-inhibitory peptides into clinically applicable therapeutic agents remains challenging, despite increasing evidence of ACE-inhibitory activity in vitro and in vivo models. Translating a potent ACE-inhibitory peptide into a clinically effective drug requires not only pharmacokinetic properties but also strong enzymatic activity, bioavailability, safety, scalable production, and reproducible efficacy in humans [132]. Current evidence remains dominated by in vitro and preclinical studies, whereas well-designed human clinical trials evaluating marine-derived ACE-inhibitory peptides remain limited [132].

Limited oral bioavailability is a major obstacle to applying marine-derived peptides. Peptides are exposed to gastric acid and intestinal proteases, which can hydrolyze the peptide sequence before sufficient quantities reach systemic circulation after oral administration. Thus, an ACE-inhibitory effect demonstrated in an in vitro assay may not be reproduced in animal models. Evaluation of gastrointestinal and plasma stability, along with pharmacokinetic strategies, should balance ACE-inhibitory activity measurements. These strategies enhance bioavailability and improve resistance to enzymatic and protein degradation [132,133].

### 9.2. Pharmacokinetic and Pharmacodynamic Obstacles

In particular, the pharmacological performance of a peptide depends on its ADME and ability to reach sufficient concentrations at the site of action. A low IC_50_ value from enzymatic bioassays does not necessarily indicate therapeutic efficacy in humans. Integrating systematic pharmacokinetic and pharmacodynamic investigations is required to establish the relationship between administered dose and ACE inhibition. In addition, the active species responsible for the biological effect may differ from the originally recognized peptide because metabolic processing can generate shorter peptide fragments. Furthermore, identifying circulating metabolites and their ACE-inhibition mechanisms is crucial for clinical development [106,132].

### 9.3. Toxicity, Allergenicity, and Immunogenicity

The potential toxicity, allergenicity, immunogenicity, and interactions with biological targets must be investigated, since marine-derived peptides of natural origin are not inherently clinically safe. This is particularly important for marine species producing allergenic protein-associated compounds. This is because long-term administration may be required for antihypertensive therapy, which is generally intended for chronic use. Recent studies also show that comprehensive safety information, including toxicity, is limited for many marine-derived antihypertensive peptides [63].

### 9.4. Isolation, Purification, and Industrial Production

Reproducible, scalable production is essential for developing marine-derived peptides as drug-discovery candidates. Marine species can vary in peptide composition depending on species and environmental conditions. However, obtaining a consistent peptide profile from a natural marine source can be challenging. Obtaining a peptide at laboratory scale does not necessarily guarantee economically viable production at pharmaceutical scale [132]. Large-scale extraction and purification must provide high purity and batch-to-batch consistency while minimizing organic solvent use. To overcome these challenges, enzyme hydrolysis, microbial fermentation, recombinant production, and chemical peptide synthesis provide alternative methods with direct extraction [134].

### 9.5. Limited Clinical Studies

The most significant obstacle is the limited availability of human clinical evidence. Although most marine peptides have been expressed in vitro and in animal models, relatively few studies have progressed to human intervention trials. Recent evidence has identified only a limited number of clinical studies, highlighting the need for broader evaluation of long-term safety effects. Therefore, clinical development requires well-designed randomized controlled trials to establish the efficacy, pharmacokinetics, and potential interactions of marine-derived ACE-inhibitory peptides. Regulatory classification may also vary by application. However, each application needs appropriate evidence regarding purity, safety, and efficacy [63,132].

## 10. The Limitations of Secondary Metabolites on Peptide Usage in ACE Inhibition

Marine organisms are important sources of structurally diverse secondary metabolites, including polyphenols, polyketides, alkaloids, and specialized metabolites with high pharmacological activity. Some of those metabolites are associated with cardiovascular diseases. Although they can be compared with marine-derived peptides, their development as ACE inhibitors faces several obstacles that must be considered when assessing their potential as antihypertensive agents [84,135]. Defining a clear structure–activity relationship is one major limitation to consider. Marine secondary metabolites often possess highly diverse and chemically complex scaffolds, including multiple functional groups. However, this structural diversity can provide pathways for discovering novel ACE-binding molecules; it can also be difficult to identify the specific structural characteristics associated with ACE inhibition. In contrast, peptide sequences provide a more readily interpretable framework in which amino acids alone and peptide length can be modified to investigate their contributions to ACE inhibition [104].

Underlying ACE inhibition, peptides can interact with the ACE Zn^2+^ catalytic channel through a combination of intermolecular interactions that determine their binding orientation. Structural characteristics can then be investigated using computational molecular methods, including molecular docking [136]. This is more challenging because the relationship between molecular structure and ACE inhibition may involve complex physicochemical and stereochemical determinants, and because experimentally modifying structural components individually is difficult. The molecular size and physicochemical characteristics of secondary metabolites may also influence the interaction with the ACE active site [136]. Some secondary metabolites strongly inhibit the enzyme by binding to substrate sites, whereas others act through nonspecific interactions not directly related to competitive occupation of the ACE catalytic channel. To attribute an observed reduction in ACE activity to specific inhibition, it should be supported by mechanisms that distinguish specific ACE inhibition from nonspecific effects [137].

Secondary metabolites undergo extensive metabolism after oral administration, which differs from the parent compound tested in vitro and affects physiological efficacy. However, marine-derived peptides face similar challenges with gastrointestinal degradation and bioavailability. Optimized sequences through substitution, modification, and other peptide-engineering strategies can enhance pharmacological properties. Additional challenges may occur during the production and standardization of marine secondary metabolites. Depending on the species and environmental conditions, concentration and yield can vary. Isolating a single metabolite requires extraction and purification methods. Similarly, peptides obtained from marine protein hydrolysates can also show batch-to-batch variability [13]. For drug discovery, marine secondary metabolites and peptides should be considered complementary sources of ACE-inhibitory compounds. Secondary metabolites provide chemically diverse scaffolds that can lead to novel mechanisms. Peptides can also offer new pathways for structure–activity studies and sequence-based optimization [84].

## 11. AI Intervention in the Discovery

Artificial intelligence (AI) and machine learning for ACE-inhibitory peptide discovery have advanced from conventional sequence-based prediction to integrated deep learning and protein-language model approaches. These approaches provide valuable computational tools to accelerate screening of marine-derived peptide sequences and reduce the experimental methods associated with conventional peptide identification [138].

In 2019, mAHTPred employed multiple sequence descriptors and an ensemble of machine learning algorithms, including random forest and support vector machine models, to improve the prediction of antihypertensive peptides. The integrated prediction model showed improved predictive performance compared with existing approaches. It also highlights the potential of machine learning to prioritize peptide candidates before experimental validation [139]. Machine learning-based QSAR modeling was specifically demonstrated for ACE-inhibitory peptides using a curated dataset containing 728 unique peptides to determine IC_50_ values. The resulting models identified structural features associated with ACE inhibition and predicted novel dipeptides [140].

More recently, deep learning approaches have improved how peptide sequence information is represented. The pLM4ACE model introduced protein-language model representations generated using ESM-2 embeddings for ACE-inhibitor peptide prediction. Among the evaluated approaches, the best model demonstrated the potential of protein-language models to identify ACE-inhibitory peptide candidates from a large sequence space [141]. The AI4ACEIP framework combined sequence information, protein-language model features, and molecular representations within a two-layer stacked ensemble-learning architecture [142]. Deep stack-ACE further advanced ACE-inhibitory peptide prediction by connecting Word2Vec sequence representations with deep learning methods, including LSTM and CNNs [143]. More recent work has moved toward computer- and AI-assisted peptide design and target optimization, introducing deepAngioin. It is a computer-assisted strategy that integrates ACE-inhibitory peptide preparation and interaction, aiming to address limitations associated with low yield and extensive experimental optimization [144]. AI can simplify the discovery of marine-derived ACE-inhibitory peptides through in silico generation and AI-based prediction, followed by molecular simulations and experimental validation. This approach reduces the need for experimental validation, but potential biases in training data and limited accounting for complex biological factors remain [145].

## 12. Future Perspectives and Conclusions

Exploring underutilized marine species to discover new antihypertensive peptides should use novel high-throughput screening and improved chromatographic techniques to improve synthesis efficiency and bioactivity. Beyond human clinical trials, data must be up to date to substantiate the biosafety, effectiveness, and bioavailability of ACE-inhibitory peptides. As functional foods, nutraceuticals, or pharmaceuticals, ACE-inhibitory peptides can be applied while maintaining bioavailability and stability across numerous food products. Eco-friendly, sustainable hygiene practices can produce clean compounds and drive product demand. Finally, clear regulatory pathways help enhance quality, efficacy, efficiency, and safety for clinical and industrial applications [146].

AI-driven computational models predict peptide properties, accelerating the discovery of marine bioactive peptides by creating databases of sequences, structures, and activities for AI and QSAR model development. This supports the consistent development of AI and QSAR models. However, poor data quality and insufficient data in inefficient datasets can reduce AI model accuracy [147].

In real-world applications, rather than relying on a single approach, integrating 3D QSAR, molecular docking, and molecular dynamics simulations enables precise mapping of peptide residues to interactions with catalytic Zn^2+^ and surrounding active-site subsites. It also enables rational de novo design of optimized drug compounds with improved metabolic stability against gastrointestinal proteases. Future studies could investigate whether peptide selection and design should target not only ACE but also multifunctional peptides with potential for wide-ranging cardioprotective effects. Because marine-derived bioactive peptides already exhibit diverse biological activities, this supports investigating multifunctional therapeutic targets [148]. Sustainability is far more than a demand strategy on Earth today. Therefore, sustainable approaches to using marine resources could provide crucial peptides while strengthening the bioeconomy [149].

In conclusion, bioactive peptides from marine algae, fish, mollusks, shellfish, and other organisms have fewer side effects than synthetic drugs. ACE-inhibitory peptides can also be considered structurally distinct compounds for cardiovascular drug discovery. However, moving these marine peptides toward therapeutic targets requires a multidisciplinary approach that integrates computational molecular mechanistic analyses with structure–activity relationship, clinical, and translational approaches. In due course, integrating medicinal chemistry and clinical evaluation can advance marine-derived peptides from peptides to viable cardiovascular therapies.

## Figures and Tables

**Figure 1:**
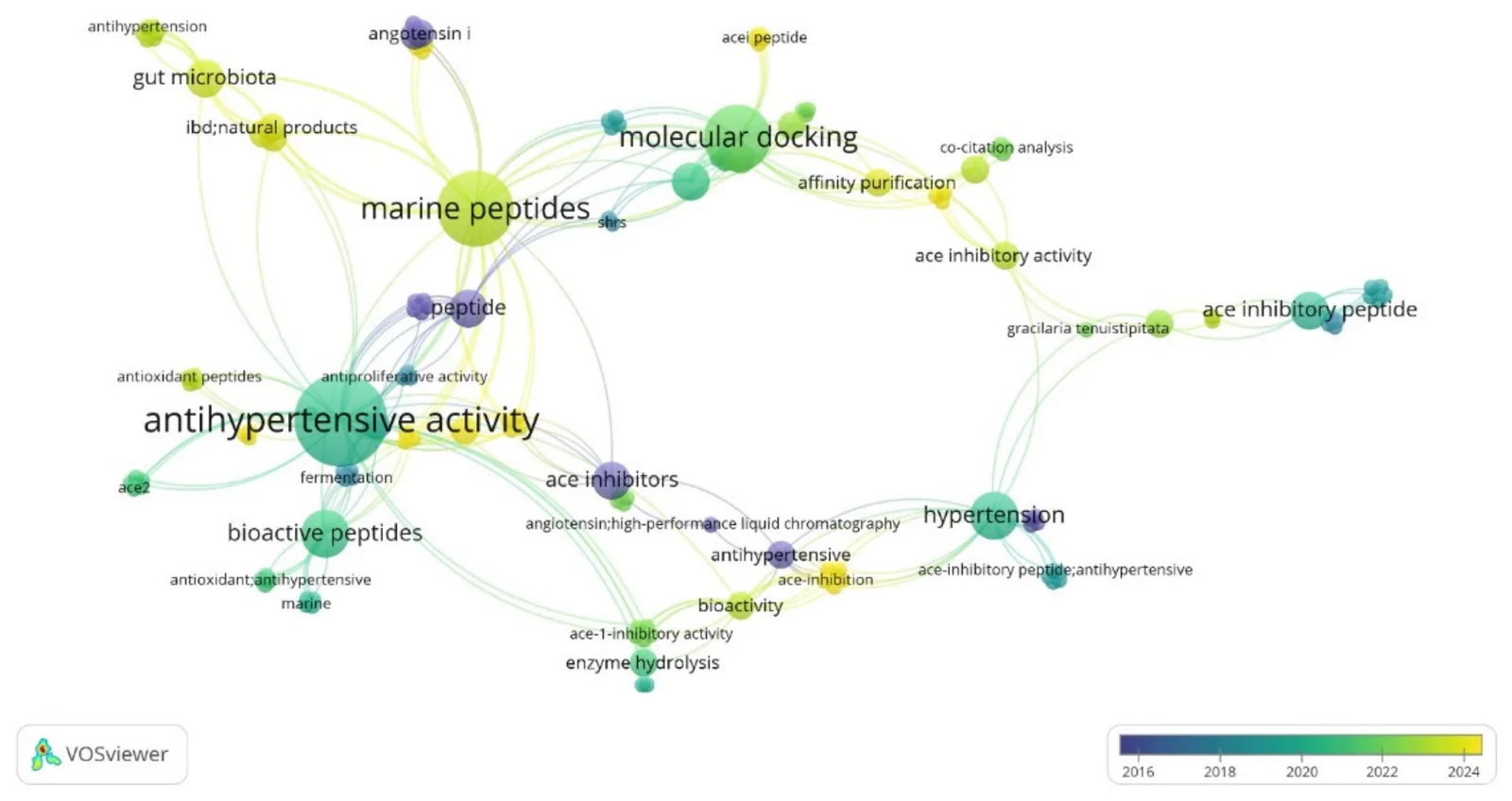
The bibliographic analysis of publications from 2016 to 2024, using VOS viewer (version 1.6.20) and Scopus data.

**Figure 2:**
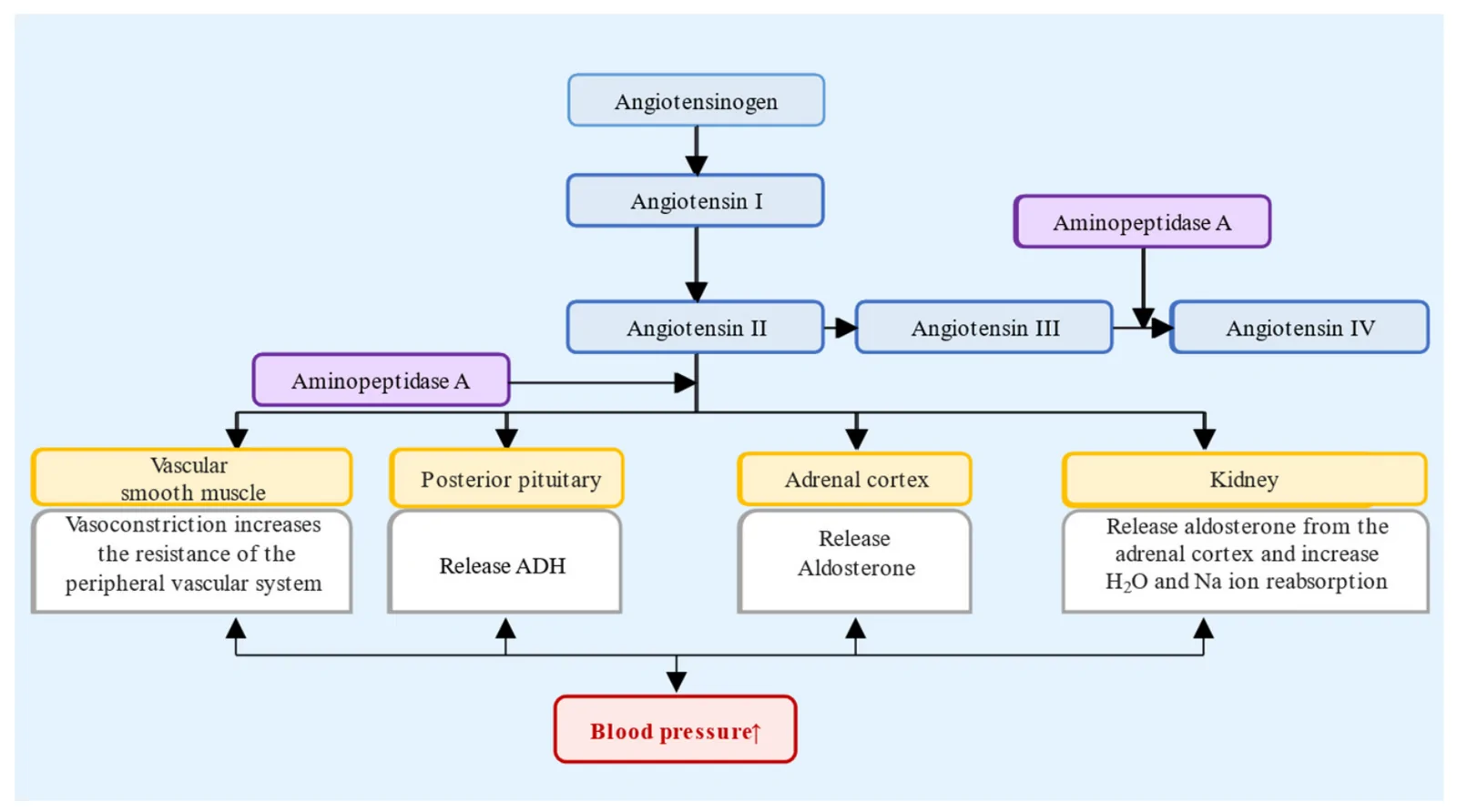
Mechanism of RAAS. The generation of hypertension within the RAAS.

**Figure 3:**
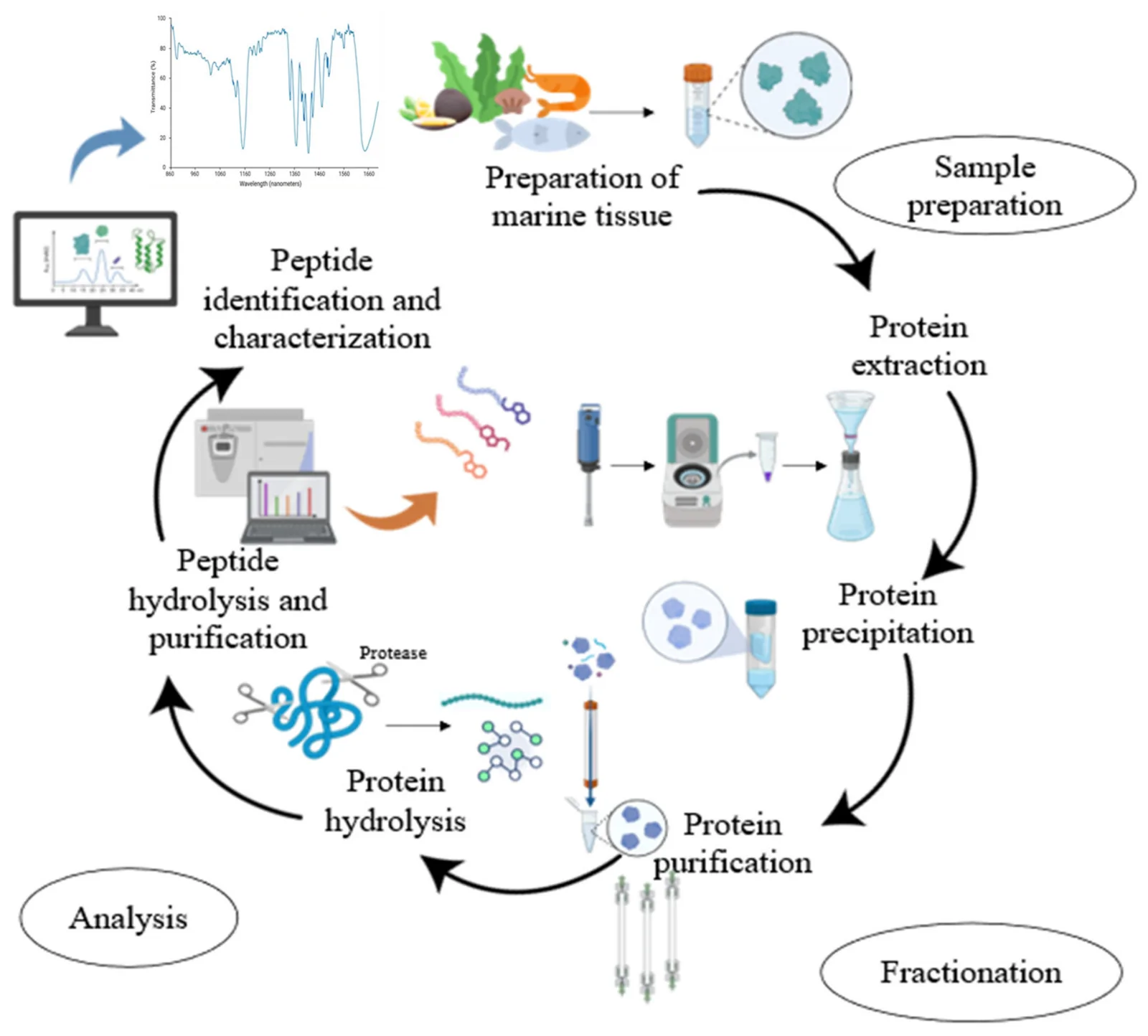
The flow diagram of the key steps of preparing ACE-inhibitory peptides.

**Figure 4:**
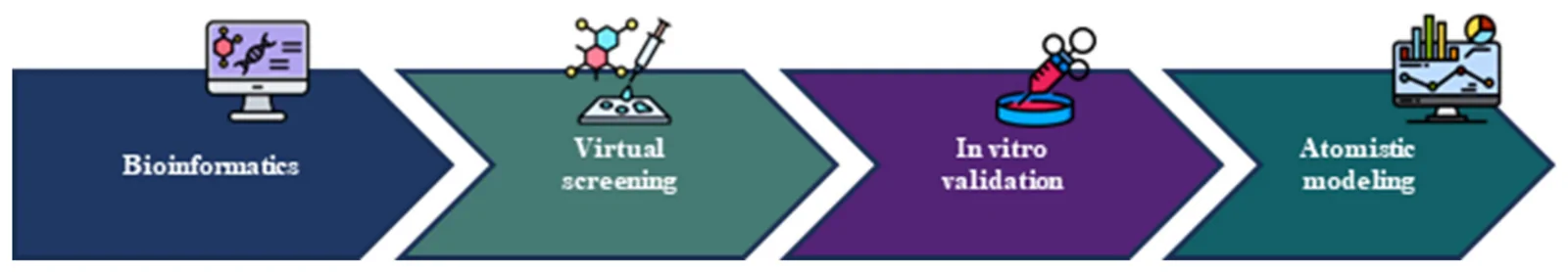
Integrated computational and experimental pipeline for discovery of marine-derived ACE-inhibitory peptides.

**Table 1:** The data on marine organisms producing bioactive proteins and peptides and their bioactivities.

Marine Source	Species Name	Peptide Sequence	Molecular Weight	Method of Extraction	Bioactivity	Reference
Seaweed	*Porphyra yezoensis*	NMEKGSSSVVSSRM	1.77 kDa	Pepsin	Anticoagulant activity	[39]
	*Palmaria palmata*	NIGK	3.5 kDa	Papain hydrolysis	Acetylhydrolase-inhibitory	[40]
		IRLIIVLMPILMA	-	Pepsin and trypsin	Renin-inhibitory	[41]
		ILAPLLAP and MAGVDHI	-	Dipeptidyl peptidase hydrolysis	Anti-diabetic	[42]
Fish	*Salmo salar*	TPEVHIAVDKF	<3 kDa	Enzyme hydrolysis	Anti-allergic	[43]
	*Sardine pilchardus*	VLGP CGSFT FNLE LLLLN CAEAGH WHSLP EVPADM NAPNPR NQGPRP DNWTF CGGWLF YACSVR TVEHVGG NAALGPR DTMYDT DWSSAPP TVCLSGGGA PVNTLPLA EVYEFDR	384.226 Da 513.178 Da 521.24 Da 584.38 Da 586.21 Da 638.31 Da 660.27 Da 667.329 Da 667.329 Da 681.26 Da 681.284 Da 697.311 Da 697.33 Da 697.376 Da 744.25 Da 758.31 Da 763.343 Da 823.469 Da 956.41 Da	Subtilisin, trypsin, and flavourzyme	Dipeptidyl peptidase IV (DPP-IV) inhibitory	[44]
	*Maurolicus muelleri* and *Meganyctiphanes norvegica combination*	-	-	Alcalase, endocut-01, endogenous M/K enzyme hydrolysis	Anti-diabetic	[45]
	Cod skin	PGEKGPSGEAGTAGPPGTPGPQGL	2163 Da	Pepsin enzyme hydrolysis	H1N1 virus neuraminidase inhibitory	[46]
	*Scomber japonicus*	LTPFT, PLITT		Papain enzyme hydrolysis	ACE-inhibitory	[47]
Shellfish	*Crassostrea gigas*	-	<2 kDa	Heat treatment	ACE-inhibitory	[48]
	*Ark shell*	LLRLTDL and GYALPCDCL	843.02 Da and 954.13 Da	Superoxide dismutase, catalase, and glutathione peroxidase hydrolysis	Anti-coagulant	[49]
	*Crassostrea talienwhanensis*	LAGELHQEQENYK AIDTIINQK DSYVGDEAQSK PGTTEDEPVK ETVIDTIQK DLESQLK NAETELGETSQR EYDESGPSIVHR DSDLEGHPTPR HDNPGDLGDLH AQCEMEPNH ESAGIHETT NTVLSGGTT	1557.74 Da 1014.57 Da 1197.52 Da 1071.51 Da 1045.57 Da 831.43 Da 1333.61 Da 1387.64 Da 1222.56 Da 1188.52 Da 1114.42 Da 943.42 Da 848.42 Da	Trypsin hydrolysis	Antioxidant	[50]
	*Mytilus coruscus*	MCPH-T1	<1 kDa	Trypsin hydrolysis	Antioxidant	[51]
	Oyster	DSQLAPFRF and HFNPRL	-	Ultrasound-assisted enzymatic hydrolysis	Antioxidant and anti-osteoporotic	[52]
	*Pinctada* *martensii*	TWP TAMY FPGA	402.1903 Da 484.1992 Da 390.1903 Da	Protease hydrolysis	Anti-inflammatory	[53]
	*Perna canaliculus*	GVKIA	<5 kDa	Pepsin and alcalase hydrolysis	ACE-inhibitory	[54]
	*Meretrix* *lusoria*	KDLEL	617.35 Da	Neutrase, protamex, alcalase, and flavourzyme	Anti-obesity and antihyperglycemic	[55]
	*Ruditapes philippinarum*	LFKKNLLTL	1089.39 Da	Trypsin hydrolysis	Cytoprotectivity	[56]
Sea cucumber	*Acaudina Molpadioides*	FLAP	<1 kDa	Papain, pepsin, trypsin, and neutrase	Antioxidant	[57]
	*Acaudina molpadioides*	-	≤1 kDa	Microwave-assisted alkaline protease hydrolysis	Antioxidant	[58]
Sea horse	*Hippocampus* *abdominalis*	IGTGIPGIW	457.26 Da	Alca pharmacological lase hydrolysis	Antioxidant	[59]
Squid	Dried squid head	REGYEK	<10 kDa	Alcalase and flavourzyme	Antioxidant ACE-inhibitory	[60]

**Table 2:** Resources for ACE-inhibitory peptides from seafood.

Seafood Type	Species	Extraction Method	Extraction Conditions	Peptide Sequence/Hydrolysate	IC_50_/Molecular Weight	References
Seaweeds	*Caulerpa lentillifera*	Enzymatic hydrolysis (thermolysin)	Enzyme-to-substrate ratio of 1:100 (*w*/*w*) for 16 h in 50 mM phosphate buffer adjusted to the optimum pH of each enzyme (thermolysin (60 °C, pH 8.5)).	FDGIP	58.89 µmol/L	[64]
	*Gracilaria tenuistipitata*	Two-step enzyme extraction (neutrase)	Enzyme-to-substrate ratio of 1:100 (*w*/*w*). Neutrase, pH 6.0, 50 °C.	MQDAITSVINAADVQGK	1776 Da	[65]
	*Isochrysis zhanjiangenis*	In vitro gastrointestinal digestion	Enzyme-to-substrate ratio: 2% (*w*/*w*), 50 °C, pH 8.5 for 4 h.	FEIHCC	61.38 µmol/L	[66]
	*Laminaria digitata*	Enzymatic hydrolysis (viscozyme)	Enzyme-to-substrate ratio: 1% (*v*/*w*), 45 °C, pH 4.5 for 4 h.	-	590 µg/mL	[35]
	*Ulva intestinalis*	Enzymatic hydrolysis (trypsin)	Enzyme-to-substrate ratio: 4% (*w*/*w*), 37 °C, pH 8.0 for 5 h.	FGMPLDR	219.35 µmol/L	[67]
	*Undaria piinnatifida*	Enzymatic hydrolysis (pepsin)	23.6 g of wakame powder with 700 mL distilled water, 45 °C, pH 2.0 for 5 h.	AIYK, YKYY, KFYG, and YNKL	213 µM, 64.2 µM, 90.5 µM, 21 µM	[68]
Fish	*Lophius litulon*	Double-enzyme system (alcalase + neutrase)	Enzyme-to-substrate ratio: 1% (*w*/*v*), for 2 h.	SEGPK	0.63 mg/mL	[64]
	*Paralichthys olivaceus*	Enzymatic hydrolysis (protamex)	Enzyme-to-substrate ratio: 5% (*w*/*v*), pH 6.0, 40 °C for 24 h.	VFSGWAA	27.50 µg/mL	[69]
	*Takifugu flavidus*	Enzymatic hydrolysis (alcalase, pepsin, and trypsin)	(Alcalase, pH 8.0, 55 °C; neutral protease, pH 7.0, 55 °C; Pepsin, pH 2.0, 37 °C) for 5 h.	PPLLFAAL	28 µmol/L	[70]
	*Thunnus thynnus*	Enzymatic hydrolysis (neutrase, alkaline)	(Solid–liquid: 1:10) and incubated at 45 °C for 3 h.	LTGCP	64.3 µmol/L	[71]
	*Trichiurus lepturus*	Enzymatic hydrolysis (alcalase)	Substrate concentration of 5.45 g/100 mL, 60 °C, and pH 8.50 under constant agitation for 4 h at 100 rpm.	QGPIGPR	81 µmol/L	[72]
Stone fish	*Actinopyga lecanora*	Enzymatic hydrolysis (bromelain)	Substrate concentration of 1/100 (*w*/*w*), 55 °C, 5.5 pH for 1 h constant agitation 150 rpm.	ALGPQFY	0.012 mmol/L	[73]
Sea cucumber	*Apostichopus japonicus*	Enzymatic hydrolysis (alcalase)	-	HDWWKER	583.6 µmol/L	[74]
Big belly seahorse	*Hippocampus abdominalis*	Enzymatic hydrolysis (alcalase)	1:20 (*w*/*v*) sample ratio, 10 mg of alcalase, pH 4.5, 37 °C for 24 h.	IGTGIPGIW	0.08 mg/mL	[75]
Kuruma shrimp	*Marsupenaeus japonicus*	Enzymatic hydrolysis (alkaline protease)	Sample ratio 1:100 (*w*/*v*), pH 10.0, 45 °C, for 1 h.	KSHPH	125.58 µM	[76]
Shellfish	*Perna canaliculus*	Enzymatic hydrolysis (pepsin)	Substrate ratio of 1:10 (*w*/*w*), pH 2.5, 37 °C for 3 h.	-	<5 kDa	[54]
Squid	*Todarodes pacificus*	Enzymatic hydrolysis (alkaline protease)	Enzyme amount: 6190 U/g, pH 10, 40 °C for 3.11 h.	FHGLPAK, IIAPPERKY, RGLPAYE, and VPSDVEF	-	[28]

**Table 3:** Clinical studies using animal models of seafood-sourced ACE-inhibitory peptides.

Species	Peptide Type	Animal Model	The Tested Concentration	The Time of the Treatment	Other Conditions	References
*Flounder*	MEVFVP	SHR	40 mg/kg of rat body weight (BW)	Blood pressure dropped at 3–6 h post-administration	Administered (oral gavage), down-regulated ET-1, Ang II, and aldosterone, while up-regulating renal p-AMPK.	[124]
*Takifugu flavidus*	PPLLFAAL	SHR	5 mg/kg BW	Short-term assay (acute/single dose response window)	Administered through intravenous injection; demonstrated noncompetitive ACE inhibition	[69]
	TLRFALHGME	SHR	4 mg/kg BW	Over 24 h	Administered using intravenous injection, ultrafiltered skin hydrolysate	[70]
*Rachycentron canadum*	IWW	SHR	56.7 mg of peptide/rat BW	Peak reduction within 2–8 h (returned to baseline by 24 h)	Administered orally	[125]
*Katsuwonus pelamis*	LKPNM	SHR	1.5 g per day	Long-term chronic administration,4–8 weeks	Administered orally	[126]
*Pacific saury*	LEPWR	SHR	2000 mg/kg BW	24 h post-incubation	Administered orally, mixed competitive ACE-inhibitory mode	[121]
*Crassostrea gigas*	AEYLCEAC	SHR	15 mg/kg BW	12–24 h, post-incubation	Administered orally, competitive ACE inhibitor mode	[127]
*Pinctada fucata*	GWA	Sprague Dawley (SD) rats	10 mg/kg BW	24 h	Administered orally as a pearl oyster meat protein hydrolysate	[66]
*Ruditapes philippinarum*	-	DOCA salt hypertensive rats	400 mg/kg BW	4–6 weeks	Administered orally using protein hydrolysate	[128]
*Hippocampus abdominalis*	CNVPLSP	SHR	100 mg/kg BW	Single oral dose, over 24 h	Administered orally	[75]
*Gracilaropsis lemaneiformis*	FQIN[M(O)] CILR	SHR	10 mg/kg BW	Monitored over 24 h	Administered orally	[67]
*Pandalus borealis*	FSYFTY	SHR	60 mg/kg BW	Monitored 2, 4, 6, 8, 24 h	Administered orally	[129]
	-	Human	1200 mg	4–8 weeks	Clinical assessment evaluating food-grade fish protein hydrolysates (FPHs) in hypertensive or pre-hypertensive volunteers	[130]
*Apostichopus japonicus*	HDWWKER	SHR	12 mg/kg	Short-term assay	Administered via intravenous injection	[71]

## Data Availability

No new data were created or analyzed in this study. Data sharing is not applicable to this article.

## Abbreviations

The following abbreviations are used in this manuscript:

2D	Two-dimensional
3D	Three-dimensional
5-FU	5-Fluorouracil
ACE	Angiotensin-converting enzyme
ADAM17	A Disintegrin and Metalloproteinase 17
APA	Aminopeptidase A
ARBs	Angiotensin receptor blockers
AT4R	Angiotensin IV receptor
CCBs	Calcium channel blockers
CVDs	Cardiovascular diseases
DNA	Deoxyribonucleic acid
DSS	Dahl salt-sensitive
EC_50_	Half-maximal effective concentration
eNOS	Endothelial nitric oxide synthase
FPH	Fish protein enzyme hydrolysates
GPC	Gel permeation chromatography
HeLa	Human cancer cell line
HPLC	High-performance liquid chromatography
IC_50_	Half-maximal inhibitory concentration
IMAM	Immobilized metal affinity medium
IRAP	Insulin-regulated aminopeptidase
KKS	Kallikrein–Kinin System
LC-HRMS	Liquid chromatography–high-resolution mass spectrometry
NMR	Nuclear magnetic resonance
MOE	Molecular operating environment
RAS	Renin–angiotensin system
SAR	Structure–activity relationship
SARS-CoV-1	Severe acute respiratory syndrome coronavirus 1
SARS-CoV-2	Severe acute respiratory syndrome coronavirus 2
SBP	Systolic blood pressure

## References

[1] Buso G., Agabiti-Rosei C., Lemoli M., Corvini F., Muiesan M.L. (2024). The Global Burden of Resistant Hypertension and Potential Treatment Options. Eur. Cardiol..

[2] Pranali M., Wandile M. (2024). CCRP1, Hypertension and Comorbidities: A Silent Threat To Global Health. Hypertens. Comorbidities.

[3] Cheraghi Z., Azmi-Naei B., Cheraghi P., Doosti-Irani A. (2025). The Global Prevalence of Uncontrolled Hypertension: A Systematic Review and Meta-Analysis. BMC Public Health.

[4] Palaniappan L.P., Allen N.B., Almarzooq Z.I., Anderson C.A.M., Arora P., Avery C.L., Baker-Smith C.M., Bansal N., Currie M.E., Earlie R.S. (2026). 2026 Heart Disease and Stroke Statistics: A Report of US and Global Data from the American Heart Association. Circulation.

[5] Mapelli M., Rubbo F.M., Costantino S., Amelotti N., Agostoni P. (2025). Pharmacological Therapy of Hfref in 2025: Navigating New Advances and Old Unmet Needs in an Eternal Balance Between Progress and Perplexities. Card. Fail Rev..

[6] van Vark L.C., Bertrand M., Akkerhuis K.M., Brugts J.J., Fox K., Mourad J.J., Boersma E. (2012). Angiotensin-Converting Enzyme Inhibitors Reduce Mortality in Hypertension: A Meta-Analysis of Randomized Clinical Trials of Renin-Angiotensin-Aldosterone System Inhibitors Involving 158,998 Patients. Eur. Heart J..

[7] Xie C., Chen R., Zhou S., Lin Y., Liu J., Su L., Pang M., Guo Z., Luo F., Chen L. (2025). Comparative Effectiveness of Angiotensin-Converting Enzyme Inhibitors Versus Angiotensin Receptor Blockers: Multidatabase Target Trial Emulation Studies. Hypertension.

[8] Du J., Xiao M., Sudo N., Liu Q. (2024). Bioactive Peptides of Marine Organisms: Roles in the Reduction and Control of Cardiovascular Diseases. Food Sci. Nutr..

[9] Okeke E.S., Okagu I.U., Chukwudozie K., Ezike T.C., Ezeorba T.P.C. (2024). Marine-Derived Bioactive Proteins and Peptides: A Review of Current Knowledge on Anticancer Potentials, Clinical Trials, and Future Prospects. Nat. Product Commun..

[10] Tang C.D., Cheng J.H., Sun D.W. (2025). Structure-Activity Relationships and Activity Enhancement Techniques of Marine Bioactive Peptides (MBPs). Crit. Rev. Food Sci. Nutr..

[11] Shahidi F., Saeid A. (2025). Bioactivity of Marine-Derived Peptides and Proteins: A Review. Mar. Drugs.

[12] Gunasekara D., Wijerathne H., Wang L., Kim H.S., Sanjeewa K.K.A. (2025). Marine Bioactive Peptides in the Regulation of Inflammatory Responses: Current Trends and Future Directions. Proteomes.

[13] Ali F., Li D., Su Y., Chen L., Cheng X., Zheng X., Mao J. (2025). Bio-Active Peptides from Marine Sources: Mechanistic Insights into Immune Regulation, Microbiota Modulation, and Intestinal Barrier Protection. Int. J. Mol. Sci..

[14] Chamata Y., Watson K.A., Jauregi P. (2020). Whey-Derived Peptides Interactions With ACE By Molecular Docking as a Potential Predictive Tool of Natural ACE Inhibitors. Int. J. Mol. Sci..

[15] Kurnianto M.A., Defri I., Syahbanu F., Aulia S.S. (2024). Fish-Derived Bioactive Peptide: Bioactivity Potency, Structural Characteristics, and Conventional and Bioinformatics Approaches for Identification. Future Foods.

[16] Giuliani M.E., Bigossi G., Lai G., Marcozzi S., Brunetti D., Malavolta M. (2024). Marine Compounds and Age-Related Diseases: The Path from Pre-Clinical Research To Approved Drugs for the Treatment of Cardiovascular Diseases and Diabetes. Mar. Drugs.

[17] Chen R., Miao Y., Hao X., Gao B., Ma M., Zhang J.Z.H., Wang R., Li S., He X., Zhang L. (2021). Investigation on the Characteristics and Mechanisms of ACE Inhibitory Peptides By a Thorough Analysis of All 8000 Tripeptides Via Binding Free Energy Calculation. Food Sci. Nutr..

[18] Manoharan S., Shuib A.S., Abdullah N. (2017). Structural Characteristics and Antihypertensive Effects of Angiotensin-I-Converting Enzyme Inhibitory Peptides in the Renin-Angiotensin and Kallikrein Kinin Systems. Afr. J. Tradit. Complement. Altern. Med..

[19] Valentini A., Heilmann R.M., Kuhne A., Biagini L., De Bellis D., Rossi G. (2025). The Renin-Angiotensin-Aldosterone System (RAAS): Beyond Cardiovascular Regulation. Vet. Sci..

[20] Rodrigues R., Costa de Oliveira S. (2021). The Impact of Angiotensin-Converting Enzyme 2 (ACE2) Expression Levels in Patients With Comorbidities on COVID-19 Severity: A Comprehensive Review. Microorganisms.

[21] Duan H., Liu G., Liu J., Chang X., Bao S., Song W., Yan W. (2024). Research Progress on Active Peptides in Marine Fish. Food Sci. Anim. Prod..

[22] Vargas Vargas R.A., Varela Millan J.M., Fajardo Bonilla E. (2022). Renin-Angiotensin System: Basic and Clinical Aspects-A General Perspective. Endocrinol. Diabetes Nutr. (Engl. Ed.).

[23] Forster P., Wysocki J., Abedini Y., Muller T., Ye M., Ferrario C.M., Batlle D. (2025). Aminopeptidase a Effect on Angiotensin Peptides and Their Blood Pressure Action. Int. J. Mol. Sci..

[24] Nehme A., Zouein F.A., Zayeri Z.D., Zibara K. (2019). An Update on the Tissue Renin Angiotensin System and Its Role in Physiology and Pathology. J. Cardiovasc. Dev. Dis..

[25] Bernstein K.E. (2006). Views of the Renin-Angiotensin System: Brilling, Mimsy, and Slithy Tove. Hypertension.

[26] Chhaniyara R.K., Gamit D.A. (2024). An Angiotensin-Converting Enzyme Inhibition Activity By Various Natural Approaches: A Statistic Review. Arch. Med. Health Sci..

[27] Allegranzi B., Tartari E., Pittet D. (2021). “Seconds Save Lives—Clean Your Hands”: The 5 May 2021 World Health Organization SAVE LIVES: Clean Your Hands campaign. J. Hosp. Infect..

[28] Li M., Liang Q., Zhang Y., Jiang X., Gu Y., Song X., Wang X., Shi W. (2025). Screening of Potential Angiotensin-Converting Enzyme-Inhibitory Peptides in Squid (*Todarodes pacificus*) Skin Hydrolysates: Preliminary Study of Its Mechanism of Inhibition. Mar. Drugs.

[29] Na Takuathung M., Sakuludomkan W., Khatsri R., Dukaew N., Kraivisitkul N., Ahmadmusa B., Mahakkanukrauh C., Wangthaweesap K., Onin J., Srichai S. (2022). Adverse Effects of Angiotensin-Converting Enzyme Inhibitors in Humans: A Systematic Review and Meta-Analysis of 378 Randomized Controlled Trials. Int. J. Environ. Res. Public Health.

[30] Manoharan S. (2023). Is It Still Relevant to Discover New ACE Inhibitors from Natural Products? YES, But only with Comprehensive Approaches to Address the Patients’ Real Problems: Chronic Dry Cough and Angioedema. Molecules.

[31] Daskaya-Dikmen C., Yucetepe A., Karbancioglu-Guler F., Daskaya H., Ozcelik B. (2017). Angiotensin-I-Converting Enzyme (ACE)-Inhibitory Peptides from Plants. Nutrients.

[32] Cheng X., Tang Z., Li Z., Hu S., Qin K., Zhao Y., Hao B., Hu Q. (2026). Marine-Derived Products as Pharmaceutical Treasure Troves: A Focus on Recent Research Techniques and Potential Bioactive Activities of Marine Peptides. Chin. J. Nat. Med..

[33] Chen J., Lv Z., Guo M. (2022). Research Advancement of Apostichopus Japonicus from 2000 to 2021. Front. Mar. Sci..

[34] Ogundele A.V., Nongthombam G.S., Nwagu A.D., Silva H.H., Fabiyi O.A. (2026). Venom-Derived Enzyme Inhibitors as Anticancer Agents: Structure-Activity Relationships, Molecular Targets and Mechanistic Insights. Molecules.

[35] Amado I.R., Vazquez J.A., Gonzalez P., Esteban-Fernandez D., Carrera M., Pineiro C. (2014). Identification of the Major ACE-Inhibitory Peptides Produced By Enzymatic Hydrolysis of a Protein Concentrate from Cuttlefish Wastewater. Mar. Drugs.

[36] Gomis-Ruth F.X., Stocker W. (2022). Structural and Evolutionary Insights into Astacin Metallopeptidases. Front. Mol. Biosci..

[37] Hamed I., Özogul F., Özogul Y., Regenstein J.M. (2015). Marine Bioactive Compounds and Their Health Benefits: A Review. Compr. Rev. Food Sci. Food Saf..

[38] Kundam D.N., Acham I.O., Girgih A.T. (2018). Bioactive Compounds in Fish and Their Health Benefits. Asian Food Sci. J..

[39] Indumathi P., Mehta A. (2016). A Novel Anticoagulant Peptide from the Nori Hydrolysate. J. Funct. Foods.

[40] Fitzgerald C., Gallagher E., O’Connor P., Prieto J., Mora-Soler L., Grealy M., Hayes M. (2013). Development of a Seaweed Derived Platelet Activating Factor Acetylhydrolase (PAF-AH) Inhibitory Hydrolysate, Synthesis of Inhibitory Peptides and Assessment of Their Toxicity Using the Zebrafish Larvae Assay. Peptides.

[41] Fitzgerald C., Aluko R.E., Hossain M., Rai D.K., Hayes M. (2014). Potential of a Renin Inhibitory Peptide from the Red Seaweed Palmaria Palmata as a Functional Food Ingredient Following Confirmation and Characterization of a Hypotensive Effect in Spontaneously Hypertensive Rats. J. Agric. Food Chem..

[42] Harnedy P.A., O’Keeffe M.B., FitzGerald R.J. (2015). Purification and Identification of Dipeptidyl Peptidase (DPP) IV Inhibitory Peptides from the Macroalga *Palmaria palmata*. Food Chem..

[43] Wang K., Siddanakoppalu P.N., Ahmed I., Pavase T.R., Lin H., Li Z. (2020). Purification and Identification of Anti-Allergic Peptide from Atlantic Salmon (*Salmo salar*) Byproduct Enzymatic Hydrolysates. J. Funct. Foods.

[44] Rivero-Pino F., Espejo-Carpio F.J., Guadix E.M. (2020). Production and Identification of Dipeptidyl Peptidase IV (DPP-IV) Inhibitory Peptides from Discarded *Sardine pilchardus* Protein. Food Chem..

[45] Naik A.S., Whitaker R.D., Albrektsen S., Solstad R.G., Thoresen L., Hayes M. (2021). Mesopelagic Fish Protein Hydrolysates and Extracts: A Source of Novel Anti-Hypertensive and Anti-Diabetic Peptides. Front. Mar. Sci..

[46] Li J., Chen Y., Yuan N., Zeng M., Zhao Y., Yu R., Liu Z., Wu H., Dong S. (2018). A Novel Natural Influenza a H1N1 Virus Neuraminidase Inhibitory Peptide Derived from Cod Skin Hydrolysates and Its Antiviral Mechanism. Mar. Drugs.

[47] Wang B., Zhang H., Wen Y., Yuan W., Chen H., Lin L., Guo F., Zheng Z.P., Zhao C. (2024). The Novel Angiotensin-I-Converting Enzyme Inhibitory Peptides from *Scomber japonicus* Muscle Protein Hydrolysates: QSAR-based screening, molecular docking, kinetic and stability studies. Food Chem..

[48] Guo Z., Zhao F., Chen H., Tu M., Tao S., Wang Z., Wu C., He S., Du M. (2020). Heat Treatments of Peptides from Oyster (*Crassostrea gigas*) and the Impact on Their Digestibility and Angiotensin I Converting Enzyme Inhibitory Activity. Food Sci. Biotechnol..

[49] Ahn C.-B., Je J.-Y. (2021). Anti-Adipogenic Peptides from Ark Shell Protein Hydrolysate: Purification, Identification and Anti-Adipogenic Effect. Process Biochem..

[50] Wang X., Yu H., Xing R., Liu S., Chen X., Li P. (2020). Optimization of Oyster (*Crassostrea talienwhanensis*) Protein Hydrolysates Using Response Surface Methodology. Molecules.

[51] Zhang Z., Jiang S., Zeng Y., He K., Luo Y., Yu F. (2020). Antioxidant Peptides from *Mytilus coruscus* on H_2_O_2_-Induced Human Umbilical Vein Endothelial Cell Stress. Food Biosci..

[52] Quan Z., Wang Z., Wang Z., Hou Z., Liu B., Guo X., Zhu B., Hu Y. (2025). Study on the Antioxidant and Antiosteoporotic Activities of the Oyster Peptides Prepared By Ultrasound-Assisted Enzymatic Hydrolysis. Ultrason. Sonochem..

[53] Shen J., Zeng M., Huang P., Chen B., Xia Z., Cao Y., Miao J. (2023). Purification and Activity Evaluation of Novel Anti-inflammatory Peptides from Pearl Oyster (*Pinctada martensii*) Hydrolysates. Food Funct..

[54] Jayaprakash R., Perera C.O. (2020). Partial Purification and Characterization of Bioactive Peptides from Cooked New Zealand Green-Lipped Mussel (*Perna canaliculus*) Protein Hydrolyzates. Foods.

[55] Kim M.J., Chilakala R., Jo H.G., Lee S.J., Lee D.S., Cheong S.H. (2022). Anti-Obesity and Anti-Hyperglycemic Effects of *Meretrix lusoria* Protamex Hydrolysate in Ob/Ob Mice. Int. J. Mol. Sci..

[56] Jin H., Zhao H., Shi R., Fan F., Cheng W. (2024). Unlocking the Therapeutic Potential of a Manila Clam-Derived Antioxidant Peptide: Insights into Mechanisms of Action and Cytoprotective Effects Against Oxidative Stress. Foods.

[57] Jin H.X., Xu H.P., Li Y., Zhang Q.W., Xie H. (2019). Preparation and Evaluation of Peptides With Potential Antioxidant Activity By Microwave Assisted Enzymatic Hydrolysis of Collagen from Sea Cucumber *Acaudina molpadioides* Obtained from Zhejiang Province in China. Mar. Drugs.

[58] Li Y., Li J., Lin S.J., Yang Z.S., Jin H.X. (2019). Preparation of Antioxidant Peptide By Microwave- Assisted Hydrolysis of Collagen and Its Protective Effect Against H(2)O(2)-Induced Damage of RAW264.7 Cells. Mar. Drugs.

[59] Lee H.G., Nagahawatta D.P., Yang F., Jayawardhana H., Liyanage N.M., Lee D.S., Lee J.M., Yim M.J., Ko S.C., Kim J.Y. (2023). Antioxidant Potential of Hydrolysate-Derived Seahorse (*Hippocampus abdominalis*) Peptide: Protective Effects Against AAPH-Induced Oxidative Damage In Vitro and In Vivo. Food Chem..

[60] Sukkhown P., Pirak T., Jangchud K., Prinyawiwatkul W. (2021). Novel Peptides from Dried Squid Head By-Products Obtained from Snack Process. Int. J. Food Sci. Technol..

[61] Vásquez P., Zapata J.E., Chamorro V.C., García Fillería S.F., Tironi V.A. (2022). Antioxidant and Angiotensin I-Converting Enzyme (ACE) Inhibitory Peptides of Rainbow Trout (*Oncorhynchus mykiss*) Viscera Hydrolysates Subjected To Simulated Gastrointestinal Digestion and Intestinal Absorption. LWT.

[62] Zhang R., Ren Y., Ren T., Yu Y., Li B., Zhou X. (2025). Marine-Derived Antioxidants: A Comprehensive Review of Their Therapeutic Potential in Oxidative Stress-Associated Diseases. Mar. Drugs.

[63] Jo D.M., Khan F., Park S.K., Ko S.C., Kim K.W., Yang D., Kim J.Y., Oh G.W., Choi G., Lee D.S. (2024). From Sea To Lab: Angiotensin I-Converting Enzyme Inhibition By Marine Peptides-Mechanisms and Applications. Mar. Drugs.

[64] Hu Y.D., Xi Q.H., Kong J., Zhao Y.Q., Chi C.F., Wang B. (2023). Angiotensin-I-Converting Enzyme (ACE)-Inhibitory Peptides from the Collagens of Monkfish (*Lophius litulon*) Swim Bladders: Isolation, Characterization, Molecular Docking Analysis and Activity Evaluation. Mar. Drugs.

[65] Su Y.-J., Liao H.-J., Yang J.-I. (2022). Purification and Identification of an ACE-Inhibitory Peptide from *Gracilaria tenuistipitata* Protein Hydrolysates. Processes.

[66] Liu P., Lan X., Yaseen M., Wu S., Feng X., Zhou L., Sun J., Liao A., Liao D., Sun L. (2019). Purification, Characterization and Evaluation of Inhibitory Mechanism of ACE Inhibitory Peptides from Pearl Oyster (*Pinctada fucata martensii*) Meat Protein Hydrolysate. Mar. Drugs.

[67] Deng Z., Liu Y., Wang J., Wu S., Geng L., Sui Z., Zhang Q. (2018). Antihypertensive Effects of Two Novel Angiotensin I-Converting Enzyme (ACE) Inhibitory Peptides from *Gracilariopsis lemaneiformis* (Rhodophyta) in Spontaneously Hypertensive Rats (Shrs). Mar. Drugs.

[68] Suetsuna K., Maekawa K., Chen J.R. (2004). Antihypertensive Effects of *Undaria pinnatifida* (wakame) Peptide on Blood Pressure in Spontaneously Hypertensive Rats. J. Nutr. Biochem.

[69] Ko J.-Y., Lee J.-H., Sim H.-H., Kim H.J., Oak M.-H. (2023). Isolation and Activity Evaluation of Peptides With Anti-Hypertensive Activity from Commercial Enzymatic Flounder Fish Hydrolysate. Nat. Product Sci..

[70] Su Y., Chen S., Cai S., Liu S., Pan N., Su J., Qiao K., Xu M., Chen B., Yang S. (2021). A Novel Angiotensin-I-Converting Enzyme (ACE) Inhibitory Peptide from *Takifugu*. Flavidus. Mar. Drugs.

[71] Wang S., Zhang L., Wang H., Liu J., Hu Y., Tu Z. (2024). Angiotensin Converting Enzyme (ACE) Inhibitory Peptide from The Tuna (*Thunnus thynnus*) Muscle: Screening, Interaction Mechanism and Stability. Int. J. Biol. Macromol..

[72] Cao J., Xiang B., Dou B., Hu J., Zhang L., Kang X., Lyu M., Wang S. (2024). Novel Angiotensin-Converting Enzyme-Inhibitory Peptides Obtained from *Trichiurus lepturus*: Preparation, Identification and Potential Antihypertensive Mechanism. Biomolecules.

[73] Sadegh Vishkaei M., Ebrahimpour A., Abdul-Hamid A., Ismail A., Saari N. (2016). Angiotensin-I Converting Enzyme (ACE) Inhibitory and Anti-Hypertensive Effect of Protein Hydrolysate from *Actinopyga lecanora* (Sea Cucumber) in Rats. Mar. Drugs.

[74] Wang Y., Chen S., Shi W., Liu S., Chen X., Pan N., Wang X., Su Y., Liu Z. (2024). Targeted Affinity Purification and Mechanism of Action of Angiotensin-Converting Enzyme (ACE) Inhibitory Peptides from Sea Cucumber Gonads. Mar. Drugs.

[75] Lee H.G., Kim H.S., An H., Baek K., Lee J.M., Yim M.J., Ko S.C., Kim J.Y., Oh G.W., Je J.G. (2022). Antihypertensive Effects of IGTGIPGIW Peptide Purified from *Hippocampus abdominalis*: P-Enos and P-AKT Stimulation in EA.Hy926 Cells and Lowering of Blood Pressure in SHR Model. Mar. Drugs.

[76] Zhou J., Han Q., Koyama T., Ishizaki S. (2023). Preparation, Purification and Characterization of Antibacterial and ACE Inhibitory Peptides from Head Protein Hydrolysate of Kuruma Shrimp, *Marsupenaeus japonicus*. Molecules.

[77] Wang X., Yu H., Xing R., Li P. (2017). Characterization, Preparation, and Purification of Marine Bioactive Peptides. BioMed Res. Int..

[78] Chen M., Li D., Zeng W., Mu J., Wang S., Liang Y. (2026). Next-Generation Bioactive Peptides from Edible Microorganisms: From AI-Driven Discovery to Sustainable Bio-Manufacturing. J. Future Foods.

[79] Zhang Y., Chen M., Bruner S.D., Ding Y. (2018). Heterologous Production of Microbial Ribosomally Synthesized and Post-Translationally Modified Peptides. Front. Microbiol..

[80] Sadeeq M., Li Y., Wang C., Hou F., Zuo J., Xiong P. (2025). Unlocking the Power of Antimicrobial Peptides: Advances in Production, Optimization, and Therapeutics. Front. Cell. Infect. Microbiol..

[81] Liang Q., Liu Z., Xu M., Zhu J., Liang Z., Zhu C., Mou H. (2025). Heterologous Expression of a Recombinant ACE Inhibitory Peptide LYPVK and Its Potential Antihypertensive Action Mechanism. Int. J. Biol. Macromol..

[82] Gomes C., Ferreira D., Carvalho J.P.F., Barreto C.A.V., Fernandes J., Gouveia M., Ribeiro F., Duque A.S., Vieira S.I. (2020). Current Genetic Engineering Strategies for the Production of Antihypertensive ACEI Peptides. Biotechnol. Bioeng..

[83] Zheng Y., Zhang Y., San S. (2020). Efficacy of a Novel ACE-Inhibitory Peptide from *Sargassum maclurei* in Hypertension and Reduction of Intracellular Endothelin-1. Nutrients.

[84] Pujiastuti D.Y., Ghoyatul Amin M.N., Alamsjah M.A., Hsu J.L. (2019). Marine Organisms as Potential Sources of Bioactive Peptides That inhibit the Activity of Angiotensin I-Converting Enzyme: A Review. Molecules.

[85] Yang W., Zou X., Zhang T., Liu Q., Liu Z., Li F., Luo Y., Wang Y., Qiu Z., Zhang B. (2025). Novel ACE-Inhibitory Peptides from Royal Jelly Proteins: Comprehensive Screening, Mechanistic Insights, and Endothelial Protection. Foods.

[86] Taghizadeh M.S., Mirzapour-Kouhdasht A., Huang J.-Y. (2025). Unraveling the Structure-Activity Relationship and Molecular Mechanisms for Targeted Therapeutic Applications of Food-Derived Angiotensin-I-Converting Enzyme Inhibitory Peptides. Food Biosci..

[87] Liang Q., Liu Z., Xiao M., Zhou W., Yu D., Liu J., Wang L., Mou H. (2025). Improving Activity of ACE Inhibitory Peptide By Sequence-Based Rational Design: Properties, Function on Endothelial Cells, and Potential Antihypertensive Mechanism. Food Front..

[88] He Z., Liu G., Qiao Z., Cao Y., Song M. (2021). Novel Angiotensin-I Converting Enzyme Inhibitory Peptides Isolated from Rice Wine Lees: Purification, Characterization, and Structure-Activity Relationship. Front. Nutr..

[89] Fang L., Geng M., Liu C., Wang J., Min W., Liu J. (2019). Structural and Molecular Basis of Angiotensin-Converting Enzyme By Computational Modeling: Insights into the Mechanisms of Different Inhibitors. PLoS ONE.

[90] Xue L., Yin R., Howell K., Zhang P. (2021). Activity and Bioavailability of Food Protein-Derived Angiotensin-I-Converting Enzyme-Inhibitory Peptides. Compr. Rev. Food Sci. Food Saf..

[91] Jana P., Samanta K., Ehlers M., Zellermann E., Backer S., Stauber R.H., Schmuck C., Knauer S.K. (2023). Impact of Peptide Sequences on Their Structure and Function: Mimicking of Virus-Like Nanoparticles for Nucleic Acid Delivery. Chembiochem.

[92] Al Musaimi O. (2024). Unlocking the Potential of Retro-Inverso (RI) Peptides as Future Drug Candidates. Int. J. Pept. Res. Ther..

[93] Li J., Liu Z., Zhao Y., Zhu X., Yu R., Dong S., Wu H. (2018). Novel Natural Angiotensin Converting Enzyme (ACE)-Inhibitory Peptides Derived from Sea Cucumber-Modified Hydrolysates By Adding Exogenous Proline and a Study of Their Structure(-)Activity Relationship. Mar. Drugs.

[94] Ben Henda Y., Labidi A., Arnaudin I., Bridiau N., Delatouche R., Maugard T., Piot J.M., Sannier F., Thiery V., Bordenave-Juchereau S. (2013). Measuring Angiotensin-I Converting Enzyme Inhibitory Activity By Micro Plate Assays: Comparison Using Marine Cryptides and Tentative Threshold Determinations With Captopril and Losartan. J. Agric. Food Chem..

[95] Pangestuti R., Kim S.K. (2017). Bioactive Peptide of Marine Origin for the Prevention and Treatment of Non-Communicable Diseases. Mar. Drugs.

[96] Mbatu P.S., Toh-Boyo G.M., Ghogomu J.N., Nfor E.N., Akitsu T. (2024). Spectroscopic, Density Functional Theory and Molecular Docking Studies of Zinc (II) and Nickel (II) Complexes of N′-(Furan-2-Ylmethylene) Furan-2-Carbohydrazide as Potential Antibacterial Drug. J. Mol. Struct..

[97] Fan Y., Yu Z., Zhao W., Ding L., Zheng F., Li J., Liu J. (2020). Identification and Molecular Mechanism of Angiotensin-Converting Enzyme Inhibitory Peptides from *Larimichthys crocea* titin. Food Sci. Hum. Wellness.

[98] Ung M.H., Varn F.S., Cheng C. (2016). In Silico Frameworks for Systematic Pre-Clinical Screening of Potential Anti-Leukemia Therapeutics. Expert Opin. Drug Discov..

[99] Silva D.F., Alves C.Q., Brandao H.N., David J.M., David J.P., Silva R.L., Franchin M., Cunha T.M., Martins F.T., Oliveira C.M. (2016). Poligalen, a New Coumarin from *Polygala boliviensis*, Reduces the Release of TNF and IL-6 Independent of NF-Kb Downregulation. Fitoterapia.

[100] Yathisha U.G., Srinivasa M.G., Chandrashekara R.B., Gopalakrishna B.K., Gowda A., Salam A.A.A., Sheshappa M.B. (2025). Computational Studies of Fish Protein Hydrolysates as Human Angiotensin I-Converting Enzyme Inhibitors. J. Health Allied Sci. NU.

[101] Kan M., Ter Z.Y., Sofian-Seng N.-S., Chang L.S., Wang S., Lim S.J. (2025). Recent Advances on Bioactive Peptide Fractionation Methods. Food Bioprocess Technol..

[102] Windarto S., Lee M.-C., Nursyam H., Hsu J.-L. (2024). A Novel Phycoerythrin-Derived Peptide from *Colaconema formosanum*: Synthesis, in Vitro, and in Silico Study on Angiotensin-Converting Enzyme (ACE) Inhibitory Activity. Biocatal. Agric. Biotechnol..

[103] Zhang H., Ouyang Y., Suo Q., Chen H., Cui J., Yue Y. (2026). From Multi-Species Screening to Targeted investigation: Discovery of ACE Inhibitory Peptides in *Gigantidas platifrons* via Peptidomics, Virtual Screening, and Molecular Dynamics Simulations. Molecules.

[104] Zhang L., Huang Q., Gong W.-Y., Wang W.-J., Yang C.-T., Zhang W.-T., Sheng J., Xie J., Tian Y. (2025). Structure-Activity Relationship Analysis of the ACE Inhibitory Activity of Three Walnut Protein Hydrolysates and Elucidation of the ACE Inhibitory Mechanism of Its Derived Peptides. LWT.

[105] Iglesias J., Saen-oon S., Soliva R., Guallar V. (2018). Computational Structure-Based Drug Design: Predicting Target Flexibility. WIREs Comput. Mol. Sci..

[106] Tian W., Zhang C., Zheng Q., Hu S., Yan W., Yue L., Chen Z., Zhang C., Kong Q., Sun L. (2024). In Silico Screening of Bioactive Peptides in Stout Beer and Analysis of ACE Inhibitory Activity. Foods.

[107] Cai X., Huang M., Huang X., Liu H., Wang T., Li L., Yang W., Luo H., Lu Y. (2023). Properties of ACE Inhibitory Peptides Isolated from *Sipunculus nudus* L and a DSPE-PEG Modification for Sustained Release Anti-Hypertension Agent. Process Biochem..

[108] Amigo L., Hernandez-Ledesma B. (2020). Current Evidence on the Bioavailability of Food Bioactive Peptides. Molecules.

[109] Ma M., Di J., Wang C., Xie Y., Cui F., Zhai Y., Zhu S., Gao J. (2025). Site-Specific Pegylation of Proteins: Insights into Structural and Functional Changes. Acta Pharm. Sin. B.

[110] Yuan L., Wang K., Lei L., Zhao D., Yang H., Fang Y., Lu K. (2025). Multispectral Bioactivity Studies of N-Terminal Fatty Acid Modified Antimicrobial Peptide Andricin B. Spectrochim. Acta A Mol. Biomol. Spectrosc..

[111] Clayton D., Hanchapola I., Thomas W.G., Widdop R.E., Smith A.I., Perlmutter P., Aguilar M.I. (2015). Structural Determinants for Binding To Angiotensin Converting Enzyme 2 (ACE2) and Angiotensin Receptors 1 and 2. Front Pharmacol..

[112] Ding Y., Ting J.P., Liu J., Al-Azzam S., Pandya P., Afshar S. (2020). Impact of Non-Proteinogenic Amino Acids in the Discovery and Development of Peptide Therapeutics. Amino Acids.

[113] Abdelhedi O., Nasri M. (2019). Basic and Recent Advances in Marine Antihypertensive Peptides: Production, Structure-Activity Relationship and Bioavailability. Trends Food Sci. Technol..

[114] Michaelis L., Menten M.L., Johnson K.A., Goody R.S. (2011). The Original Michaelis Constant: Translation of the 1913 Michaelis-Menten Paper. Biochemistry.

[115] Ni H., Li L., Liu G., Hu S.Q. (2012). Inhibition Mechanism and Model of an Angiotensin I-Converting Enzyme (ACE)-Inhibitory Hexapeptide from Yeast *(Saccharomyces cerevisiae*). PLoS ONE.

[116] Lu Y., Wang Y., Huang D., Bian Z., Lu P., Fan D., Wang X. (2021). Inhibitory Mechanism of Angiotensin-Converting Enzyme Inhibitory Peptides from Black Tea. J. Zhejiang Univ. Sci. B.

[117] Theivendren P., Panneerselvam S., Swaminathan J., Murugesan V., Pradeep K., Jan Bhat M., Zarfishan A., Chidambaram K., Kiruthiga N. (2026). Marine Bacterial Metabolites in Anticancer Drug Discovery: Ecological Insights, Mechanisms of Action, and Clinical Translation. Reg. Stud. Mar. Sci..

[118] Gao N., Sun J., Li X., Yao Y., Hu Y., Zhao J., Shan A., Wang J. (2025). Overcoming Delivery Challenges of Antimicrobial Peptides for Clinical Translation: From Nanocarriers To Molecular Modifications. Drug Resist Updat.

[119] Jacob S., Nair A.B., Boddu S.H.S., Gorain B., Sreeharsha N., Shah J. (2021). An Updated Overview of the Emerging Role of Patch and Film-Based Buccal Delivery Systems. Pharmaceutics.

[120] Kulkarni N., Rao P., Jadhav G.S., Kulkarni B., Kanakavalli N., Kirad S., Salunke S., Tanpure V., Sahu B. (2023). Emerging Role of Injectable Dipeptide Hydrogels in Biomedical Applications. ACS Omega.

[121] Gunther A., Bednarczyk-Cwynar B. (2025). Oleanolic Acid: A Promising Antioxidant-Sources, Mechanisms of Action, Therapeutic Potential, and Enhancement of Bioactivity. Antioxidants.

[122] Huang Y.L., Li S.H., Chuang T.Y., Chow C.J. (2020). In Vitro Evaluation of Antioxidant Activities and Inhibition of ACE Activity By Collagenase-Treated Hydrolysate Derived from Bullfrog Skin. J. Food Drug Anal..

[123] Ko S.C., Jung W.K., Lee S.H., Lee D.H., Jeon Y.J. (2017). Antihypertensive Effect of an Enzymatic Hydrolysate from Styela Clava Flesh Tissue in Type 2 Diabetic Patients With Hypertension. Nutr. Res. Pract..

[124] Rahmdel M., Cho S.M., Jeon Y.J., Lee D.H. (2021). A Flounder Fish Peptide Shows Anti-Hypertensive Effects By Suppressing the Renin-Angiotensin-Aldosterone System and Endothelin-1. Protein Pept. Lett..

[125] Lin Y.H., Chen C.A., Tsai J.S., Chen G.W. (2019). Preparation and Identification of Novel Antihypertensive Peptides from the in Vitro Gastrointestinal Digestion of Marine Cobia Skin Hydrolysates. Nutrients.

[126] Zheng S.L., Luo Q.B., Suo S.K., Zhao Y.Q., Chi C.F., Wang B. (2022). Preparation, Identification, Molecular Docking Study and Protective Function on Huvecs of Novel ACE Inhibitory Peptides from Protein Hydrolysate of Skipjack Tuna Muscle. Mar. Drugs.

[127] Chen H., Chen Y., Zheng H., Xiang X., Xu L. (2022). A Novel Angiotensin-I-Converting Enzyme Inhibitory Peptide from Oyster: Simulated Gastro-Intestinal Digestion, Molecular Docking, Inhibition Kinetics and Antihypertensive Effects in Rats. Front. Nutr..

[128] Sun Z., Wang W., Liu J., Zou S., Yin D., Lyu C., Yu J., Wei Y. (2023). Bioactive Peptides from *Ruditapes philippinarum* Attenuate Hypertension and Cardiorenal Damage in Deoxycorticosterone Acetate-Salt Hypertensive Rats. Molecules.

[129] Gildberg A., Arnesen J.A., Sæther B.-S., Rauø J., Stenberg E. (2011). Angiotensin I-Converting Enzyme Inhibitory Activity in a Hydrolysate of Proteins from Northern Shrimp (*Pandalus borealis*) and Identification of Two Novel Inhibitory Tripeptides. Process Biochem..

[130] Musa-Veloso K., Paulionis L., Pelipyagina T., Evans M. (2019). A Randomized, Double-Blind, Placebo-Controlled, Multicentre Trial of the Effects of a Shrimp Protein Hydrolysate on Blood Pressure. Int. J. Hypertens..

[131] Malkawi R. (2026). Smart Peptide-Based Drug Delivery Systems: Stimuli-Responsive Platforms for Precision Therapeutics. J. Drug Deliv. Sci. Technol..

[132] Walquist M.J., Eilertsen K.E., Elvevoll E.O., Jensen I.J. (2024). Marine-Derived Peptides With Anti-Hypertensive Properties: Prospects for Pharmaceuticals, Supplements, and Functional Food. Mar. Drugs.

[133] Jeyavelkumaran R., Harikrishnan S., Dhanapal M.S., Keerthivasan N., Valliappan S. (2026). Engineering Peptides for Oral Delivery: Structural Design, Barrier Modulation, and Emerging Therapeutic Technologies. Pept. Sci..

[134] Agoni C., Fernandez-Diaz R., Timmons P.B., Adelfio A., Gomez H., Shields D.C. (2025). Molecular Modelling in Bioactive Peptide Discovery and Characterisation. Biomolecules.

[135] Desantis F., Miotto M., Di Rienzo L., Milanetti E., Ruocco G. (2022). Spatial Organization of Hydrophobic and Charged Residues Affects Protein Thermal Stability and Binding Affinity. Sci. Rep..

[136] Antony P., Baby B., Rahma A., Samad S.A., Dhaheri Y.A., Vijayan R. (2024). Molecular Insights into the Inhibition of Angiotensin-Converting Enzyme 1 By Hemopressin Peptides. Sci. Rep..

[137] Paiva L., Lima E., Neto A.I., Baptista J. (2017). Angiotensin I-Converting Enzyme (ACE) Inhibitory Activity, Antioxidant Properties, Phenolic Content and Amino Acid Profiles of *Fucus spiralis* L. Protein Hydrolysate Fractions. Mar. Drugs.

[138] Wang L., Niu D., Wang X., Khan J., Shen Q., Xue Y. (2021). A Novel Machine Learning Strategy for the Prediction of Antihypertensive Peptides Derived from Food With High Efficiency. Foods.

[139] Manavalan B., Basith S., Shin T.H., Wei L., Lee G. (2019). mAHTPred: A Sequence-Based Meta-Predictor for Improving the Prediction of Anti-Hypertensive Peptides Using Effective Feature Representation. Bioinformatics.

[140] Wang Y.T., Russo D.P., Liu C., Zhou Q., Zhu H., Zhang Y.H. (2020). Predictive Modeling of Angiotensin I-Converting Enzyme Inhibitory Peptides Using Various Machine Learning Approaches. J. Agric. Food Chem..

[141] Du Z., Ding X., Hsu W., Munir A., Xu Y., Li Y. (2024). pLM4ACE: A Protein Language Model Based Predictor for Antihypertensive Peptide Screening. Food Chem..

[142] Yang S., Ni J., Xu P. (2024). AI4ACEIP: A Computing Tool To Identify Food Peptides With High Inhibitory Activity for ACE By Merged Molecular Representation and Rich Intrinsic Sequence Information Based on an Ensemble Learning Strategy. J. Agric. Food Chem..

[143] Charoenkwan P., Chumnanpuen P., Schaduangrat N., Shoombuatong W. (2025). Deepstack-ACE: A Deep Stacking-Based Ensemble Learning Framework for the Accelerated Discovery of ACE Inhibitory Peptides. Methods.

[144] Yang X., Ge C., Lu L., Fan Y., Zhang J., Zhang H., Yu R., Ahmad K., Hou H. (2026). ACE Inhibitory Peptide Tailored Preparation, Screening, and Interaction Mechanism Via Computer-Assisted Experimental Studies. J. Agric. Food Chem..

[145] Qian Z., Zhou Y., Lin R., Hong Y., Xu Y., Li S., Gao J., Zhu Q., Zheng H. (2026). Deep Learning and Artificial Intelligence in the Design of Angiotensin-Converting Enzyme (ACE) Inhibitory Peptides. J. Artif. Intell. Res. Innov..

[146] Yao W., Zhang Y., Zhang G. (2024). Marine Peptides as Potential Anti-Aging Agents: Preparation, Characterization, Mechanisms of Action, and Future Perspectives. Food Chem..

[147] Mamoudou H., Mune M.A.M. (2025). AI-Driven Bioactive Peptide Discovery of Next-Generation Metabolic Biotherapeutics. Appl. Food Res..

[148] Siguenza J., Baykara H. (2026). Molecular Docking and Dynamics Simulations of 4-Heteroarylidenamino-4,5-Dihydro-1H-1,2,4-Triazol-5-one Derivatives as Potential Anticancer Agents. Comput. Biol. Chem..

[149] Nurkolis F. (2025). Marine bioactives: Pioneering Sustainable Solutions for Advanced Cosmetics and Therapeutics. Pharmacol. Res..

